# Cleaving PINK1 or PGAM5? Involvement of PARL in Methamphetamine‐Induced Excessive Mitophagy and Neuronal Necroptosis

**DOI:** 10.1111/cns.70293

**Published:** 2025-02-27

**Authors:** Di An, Chuling Zhang, Peng Zhou, Yifei Wang, Sining Meng, Yanlong Chen, Weixiao Xu, Jiankang Xuan, Jianping Xiong, Jie Cheng, Rong Gao, Jun Wang, Xufeng Chen

**Affiliations:** ^1^ Department of Emergency The First Affiliated Hospital of Nanjing Medical University Nanjing Jiangsu China; ^2^ Key Lab of Modern Toxicology, Department of Toxicology, School of Public Health, Ministry of Education Nanjing Medical University Nanjing Jiangsu China; ^3^ Key Lab of Modern Toxicology, Department of Hygienic Analysis and Detection, School of Public Health, Ministry of Education Nanjing Medical University Nanjing Jiangsu China; ^4^ China International Cooperation Center for Environment and Human Health Nanjing Medical University Nanjing Jiangsu China

**Keywords:** methamphetamine, mitophagy, necroptosis, PARL, PGAM5

## Abstract

**Background:**

Methamphetamine (Meth) is a potent psychoactive stimulant that triggers complex neurotoxicity characterized by autophagy‐associated neuronal death. However, the potential mechanisms remain poorly understood. This study aimed to decipher the Meth‐induced neuronal necroptosis involving mitochondrial defect‐initiated excessive mitophagy caused by aberrant presenilin‐associated rhomboid‐like (PARL) cleavage of PTEN‐induced kinase 1 (PINK1) and phosphoglycerate mutase family member 5 (PGAM5).

**Methods and Results:**

With the transcriptome analysis, Meth exposure significantly affected autophagy, mitophagy, and necroptosis pathways; meanwhile, the proteomic analysis revealed a marked decline in the level of PARL, which led to an imbalance in intramembrane proteolysis of PINK1 and PGAM5. In behavioral tests, Meth administration elicited pronounced cognitive decline in mice, accompanied by decreased neuronal numbers, massive autophagosomes, and mitochondrial fragmentation, and these processes can be dramatically reversed by knockin of PARL and knockdown of PGAM5 in the mouse hippocampus, molecularly manifesting as decreased necrosome formation and phosphorylated mixed lineage kinase domain‐like (p‐MLKL) mitochondrial membrane translocation, and improved autophagic flux.

**Conclusion:**

In summary, these findings collectively underscore the key roles of the PARL‐PGAM5 axis in Meth‐mediated neuronal necroptosis and that targeting this axis may provide promising therapeutic strategies for mitigating Meth‐induced neurotoxicity.

## Graphical abstract

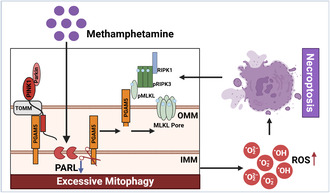

## 1 Introduction

Amphetamine‐type stimulants (ATSs) have become one of the most widely abused and highly addictive drugs in the world. According to the 2022 World Drug Report, the global ATS abusers reached approximately 34 million (UNDODC 2022), and methamphetamine (Meth) is the most typical representative of ATSs. Due to high liposolubility, it can quickly cross the blood–brain barrier (BBB) [1] and selectively act on certain brain regions, including the prefrontal cortex, striatum, and hippocampus, leading to irreversible damage and even neuronal death [2]. The hippocampus plays a pivotal role in learning and memory [3], making it a critical region of interest in neurological research. Our previous study found that Meth exposure stimulated the expression of Alzheimer's disease (AD) like pathological proteins, including amyloid‐β_42_ (Aβ_42_) and phosphorylated tau (p‐tau) [4, 5], which are commonly expressed in the hippocampus, thereby aggravating neuronal damage and underscoring the importance of selecting hippocampal neurons for this study.

Different from traditional apoptosis and autophagy, necroptosis is characterized by loss of plasma membrane integrity, swelling of organelles, and leakage of intracellular contents [6, 7, 8]. Of note, receptor‐interacting kinase‐1/3 (RIPK1/3) and mixed lineage kinase domain‐like (MLKL) are key regulators of necroptosis, which form a multiprotein complex termed the necrosome [9, 10]. Phospho‐MLKL (p‐MLKL) is a crucial player in necroptosis as it shuttles to the membrane to form pores, which contribute to cell swelling and lysis [9, 11], reactive oxygen species (ROS) generation, and inflammatory cytokines release.

Available reports have revealed that Meth‐induced neurotoxicity is closely associated with necroptosis [12, 13, 14]. It is found that necroptosis of cortical neurons occurred after Meth treatment. Besides, excessive expression of RIP3 and MLKL was observed in primary cortical neurons and postmortem brain tissues of Meth abusers [14].

The increased oxidative stress resulting from impairments of mitochondrial integrity could lead to necroptosis [15]. As a potential target organelle, the adverse effects of mitochondrial damage in Meth‐mediated neurotoxicity were recently indicated [16]. For instance, Meth induced astrocyte mitochondrial dysfunction, accompanied by mitochondrial membrane depolarization, adenosine triphosphate (ATP) depletion, and ROS generation [17]. Despite the extensive focus on mitochondrial‐mediated apoptosis [18, 19], current studies have shed light on how mitochondrial dysfunction and necroptosis play a critical role in Meth‐mediated neuronal damage. In our previous work, Meth induced the accumulation of immature autophagosomes, contributing to neuronal apoptosis and finalizing cognitive impairments [20, 21]. Nonetheless, the links between the autophagic defects and mitochondrial damage induced by Meth remain enigmatic. Mitophagy is a crucial mitochondrial quality control mechanism by eliminating damaged and redundant mitochondria [22, 23, 24]. The defective mitophagy leads to the accumulation of dysfunctional mitochondria, thereby exacerbating oxidative stress and inflammation [25]. An emerging view has implicated that mitophagic defects may play a critical role in triggering cell death [26, 27]. These results, along with our previous studies implicating autophagic defects and neuroinflammation induced by Meth [28], imply that mitophagy disturbance may be involved in Meth‐induced necroptosis; therefore, deciphering the underlying mechanisms of mitophagy defects is imperative.

Presenilin‐associated rhomboid‐like (PARL), a protease located in the inner mitochondrial membrane, regulates mitophagy by balancing intramembrane proteolysis of PTEN‐induced kinase 1 (PINK1) and phosphoglycerate mutase family member 5 (PGAM5). PGAM5 presents as two splice variants, PGAM5L (long form) and PGAM5S (short form). In polarized mitochondria, PARL cleaves PINK1, while during depolarization, PARL preferentially cleaves PGAM5 [29, 30, 31]. It has been reported that PGAM5 plays a critical role in necroptosis by facilitating mitochondrial fragmentation [32] and stabilizes PINK1 on the outer membrane of damaged mitochondria, thereby regulating PINK1/Parkin‐mediated mitophagy [33, 34]. In the current study, Meth exposure exhibits an aberrant cleavage of PGAM5 and PINK1 by PARL in neurons, causing mitochondrial damage and excessive mitophagy. Moreover, the roles of the PARL‐PGAM5 axis in mitochondrial clearance and the cognitive behaviors in mice are also examined, which might provide novel insights into potential strategies for Meth‐induced neurotoxicity.

## 2 Materials and Methods

### 2.1 Primary Neuron Cultures and Meth Treatment

Primary cultures of rat hippocampus tissues were dissected from fetal Sprague–Dawley (SD) rats (embryonic day 18), and the neurons were maintained in neurobasal medium (21103049, Gibco, USA) supplemented with 2% B27 (17504044, Gibco, USA) at 37°C in a 5% CO_2_ incubator. Half of the medium was replaced every 3 days. After 7 days, the neurons were exposed to Meth obtained from the National Institutes for Food and Drug Control (Beijing, China).

### 2.2 In Vivo Meth Administration

Male C57BL/6 mice were obtained from the Experimental Animal Center of Nanjing Medical University. The animals were housed in cages with temperature (23°C ± 2°C), humidity (45% ± 5%), and a 12 h dark/light cycle. During the experiment, the animals were provided free access to food and water. All experiments were conducted under the control of the Ethics Committee of Animal Care and Experimentation of Europe and approved by the Institutional Animal Care and Use Committee (IACUC) at Nanjing Medical University (approval no. 2302024). Meth (10 mg/kg) or saline was administered to the mice every 2–3 h in four successive intraperitoneal (i.p.) injections within 24 h. Behavior tests were performed after the last injection of Meth or saline.

### 2.3 RNA Sequencing (RNA‐Seq) and Data Analysis

RNA extracted from primary neurons was used to prepare mRNA libraries (Illumina platform, LC‐Bio Technology Co. Ltd, Hangzhou, China). After cluster generation, transcriptome sequencing was carried out on an Illumina Novaseq 6000 platform that generated raw reads. The mapped clean‐read number was normalized to RPKM (reads per kilo of per million mapped reads). The edgeR package was used to determine the StringTie genes. The threshold of significant difference was |log2foldchange| ≥ 2, *p* < 0.05. The Gene Ontology (GO) enrichment analysis, Kyoto Encyclopedia of Genes and Genomes (KEGG) pathway, Gene Set Enrichment Analysis (GSEA), and Volcano Plot analysis were conducted.

### 2.4 Proteomics

The mouse brain tissues were treated with Meth or Saline and then collected with lysis solution. The samples were homogenized using a tissue lyser followed by 15 min centrifugation (20,000 × *g*, 4°C) to collect the supernatant. Mass spectrometry analysis was performed and analyzed at LC Sciences (Hangzhou, China). Volcano Plot analysis and KEGG pathway analysis were conducted.

### 2.5 Stereotaxic Injection of Adeno‐Associated Virus

Mice were anesthetized with isoflurane and placed in a stereotactic apparatus (RWD Life Science). The rAAV‐hSyn‐PARL‐P2A‐EGFP‐WPRE‐hGH (PARL) and the rAAV‐U6‐shPGAM5‐CMV‐mCherry‐SV40 (ShPGAM5) were built by Brain VTA (Wuhan, China). The adeno‐associated virus (AAV) titer was 5.88E+12 vg/mL and the sequence of shPGAM5 was GATCTTCATATGCCATGCCAA. A total of 1.6 μL of the virus was injected bilaterally into the hippocampus of mice. The bregma and posterior coordinates were used as the reference points. To overexpress PARL or knockdown PGAM5, the virus particle was bilaterally injected in 4‐week‐old mice at the following target coordinates: anteroposterior (AP) 2.1 mm (CA1)/2.9 mm (CA3), mediolateral (ML) 1.7 mm (CA1)/3 mm (CA3), and dorsoventral (DV) 2.1 mm (CA1)/3.8 mm (CA3) from bregma. The sham‐operated mice were injected with the empty vector using the same procedure. Three weeks later, the mice underwent Meth treatment and behavioral tests.

### 2.6 Nissl Staining

The paraffin‐embedded sections were immersed in a Nissl stain solution, then the brain slides were dehydrated before being sealed with neutral balsam and imaged under a light microscope. The number of Nissl bodies in stained tissue sections was calculated, and surviving neurons were assessed based on the presence or absence of Nissl bodies.

### 2.7 Neurobehavioral Assay

For each test, mice were habituated in the testing room for 2 h. Each box was cleaned with 75% alcohol between trials to eliminate olfactory cues.

#### 2.7.1 Y Maze Test

The test was performed by naming the arms as a starter arm (SA), a familiar arm (FA), and a novel arm (NA). Each mouse was allowed to move freely in 2 arms (SA and FA) of the maze for 10 min, with the NA blocked. After an interval of 1 h, the stopper from the NA was removed, and the mice were placed in the same SA with free access to all three arms for 5 min. The duration that the mice spent on and the number of entries into each arm were analyzed for spatial recognition memory.

#### 2.7.2 Novel Object Recognition Test

In the habituation phase, each mouse was allowed to freely explore the behavioral arena with identical objects located in opposite and equidistant corners for 10 min. After a 1 h interval, the mouse was allowed to explore one initial object and one novel object with a different shape and color for 5 min in the same box. The time spent exploring each object was recorded. The recognition index was calculated using the following formula:

$$\begin{array}{c} \text{Recognition Index}=(\text{time spent}\;\mathrm{on}\;\text{novel objects}/\\ \;\text{total time spent}\;\mathrm{on}\;\text{both objects})\times 100\%. \end{array}$$

### 2.8 Adenoviral Vector Transfection

Adenoviral vectors carrying 3 × Flag‐PARL, 3 × Flag‐ShPGAM5, and mCherry‐GFP‐LC3 (HB‐AP2100001) were purchased from Hanbio (Shanghai, China), and the transfection was carried out according to the manufacturer's instructions. After adenovirus (Ad) infection for 12 h, the neurons were treated with Meth for 24 h and harvested for further analysis.

### 2.9 Transmission Electron Microscopy

The hippocampus was cut into blocks (Leica EM UC7, Germany), fixed with 2.5% glutaraldehyde overnight at 4°C, postfixed in 1% osmium tetroxide for 2 h at 37°C, and dehydrated in a graded series (20%–100%) of ethanol. The pellets were subsequently embedded in Epon 812 for 48 h at 60°C. Images were obtained by Transmission Electron Microscopy (TEM) (HT7700, Hitachi, Japan).

### 2.10 Western Blotting

The cells and tissue samples were lysed in RIPA lysis buffer containing a protease inhibitor cocktail. The protein concentrations of all samples were determined using a BCA protein assay kit (23,227, Thermo Fisher Scientific, USA). Equal amounts of protein (20 μg) were electrophoresed by 10%–12% SDS‐PAGE gradient gel (PG113, Epizyme Biotechnology, China) and transferred to PVDF membranes. Then, the membranes were blocked by 3% BSA (07‐248, Sigma‐Aldrich, Germany) for 1 h at room temperature. After that, the membranes were incubated with the primary antibody diluent at 4°C overnight and incubated with the horseradish peroxidase (HRP)‐conjugated secondary antibodies (A0208, Beyotime, China) at room temperature for 1 h. Protein bands were visualized using enzyme‐catalyzed chemiluminescence and quantified using ImageJ software. β‐actin was used as a reference. The primary antibodies used were p62 (ab109012, 1:10,000) antibody was obtained from Abcam; LC3 (12,741, 1:1000), RIP1 (3493, 1:1000), and MLKL (37,705, 1:1000) antibodies were obtained from Cell Signaling Technology; PARL (NBP1‐80878, 1:500) and PINK1 (BC100‐494, 1:1000) antibodies were obtained from Novus Biologicals; RIP3 (sc‐374,639, 1:500) and β‐actin (sc‐47,778, 1:500) antibodies were obtained from Santa Cruz Technology; PGAM5 (28445‐1‐AP, 1:2000), Fis1 (10956‐1‐AP, 1:4000), Drp1 (12957‐1‐AP, 1:2000), and Parkin (14060‐1‐AP, 1:2000) antibodies were obtained from Proteintech.

### 2.11 Lactic Dehydrogenase Release Measurement

After Meth treatment, the supernatant of neurons was collected, and Lactic Dehydrogenase (LDH) levels were detected using the LDH Activity Assay Kit (C0016, Beyotime, China) according to the manufacturer's protocol. The absorbance at 490 nm was measured with a microplate reader (Infinite M200, TECAN, China).

### 2.12 Immunofluorescence Staining

The primary neurons were fixed with 4% paraformaldehyde for 30 min and then rinsed with 0.3% Triton X‐100 for 15 min. After 5% bovine serum albumin blockage, the cells were incubated at 4°C overnight with the following primary antibodies: LC3 (12741, 1:200), RIP1 (3493, 1:200), and Tom20 (42406S, 1:200) antibodies were obtained from Cell Signaling Technology; PARL (NBP1‐80878) antibody was obtained from Novus Biologicals, RIP3 (sc‐374,639, 1:200), LC3 (sc‐398,822, 1:200), Tom20 (sc‐17,764, 1:200), and PINK1 (sc‐514,353, 1:200) antibodies were obtained from Santa Cruz Technology; p‐MLKL (ab196436, 1:200) antibody was obtained from Abcam; PGAM5 (68116‐1‐Ig, 1:1000), MLKL (66675‐1‐Ig, 1:200), RIP3 (17563‐1‐AP, 1:200), LAMP1 (67300‐1‐Ig, 1:200), and Parkin (14060‐1‐AP, 1:200) were obtained from Proteintech. Then, the cells were incubated with a fluorescently labeled secondary antibody Alexa Fluor 488 goat anti‐mouse IgG (A21206, Invitrogen, USA) or Alexa Fluor 568 goat anti‐rabbit (A10036, Invitrogen, USA) at a 1:500 ratio for 2 h, and the nuclei were stained with DAPI (C1006, Beyotime, China). Images were captured with a fluorescence microscope (LSM900, Zeiss, Germany), and Pearson's correlation was shown to evaluate the merged colocalization level by Image J software.

### 2.13 Calcein‐AM/Propidium Iodide Staining

After Meth treatments, the primary neurons were incubated in buffer containing 2 mmol/L Calcein‐AM and 1.5 mmol/L propidium iodide (PI; C2015S, Beyotime, China) at 37°C for 15 min, and the cells were observed at 488 nm excitation and 545 nm emission under fluorescence microscopy (LSM900, Zeiss, Germany). Then the proportion of dead cells was calculated using the following formula:

$$\begin{array}{cc} \text{Percentage of}\;\mathrm{PI}-\text{positive cells}= & \left(\text{number of}\;{\mathrm{PI}}^{+}\;\text{cells}\right)/\\ & \left(\text{number of Calcein}-{\mathrm{AM}}^{+}\;\text{cells}+\right.\\ & \left.\text{number of}\;{\mathrm{PI}}^{+}\;\text{cells}\right)\times 100\%. \end{array}$$

### 2.14 Measurement of Mitochondrial Reactive Oxygen Species

The Mitochondrial Reactive Oxygen Species (mtROS) was measured by MitoSOX Red (M36008, ThermoFisher Scientific, USA). The primary neurons were incubated with MitoSOX (5 μM) for 20 min at 37°C. Then the fluorescence intensity was detected by confocal microscopy (LSM900, Zeiss, Germany). The mtROS level was semi‐quantitatively analyzed by ImageJ software using the following formula:

Mean intensity=Integrated Density/Area.

### 2.15 Mitochondrial Membrane Potential Assessment

The mitochondrial membrane potential (MMP) was detected by a JC‐1 Assay Kit (C2006, Beyotime, China) according to the instructions of the manufacturer. Briefly, the primary neurons were incubated with JC‐1 working solution for 20 min, then washed with JC‐1 staining buffer, and the images were captured under a fluorescence confocal microscope (LSM900, Zeiss, Germany), and the ratio of JC‐1 aggregates to monomers was detected.

### 2.16 Mitotracker Staining

Mitotracker Red CMXRos (C1049B, Beyotime, China) was used to label mitochondria according to the manufacturer's instructions. In brief, the neurons were incubated with Mitotracker Red working solution (100 nM) at 37°C for 20 min and subjected to confocal microscopy (LSM900, Zeiss, Germany). The length of mitochondria was determined and calculated by ImageJ software.

### 2.17 Enzyme‐Linked Immunosorbent Assay

After Meth treatment, the culture medium of primary neurons was obtained, and the tumor necrosis factor‐α (TNF‐α) was detected by an Enzyme‐Linked Immunosorbent Assay (ELISA) kit (MM‐0180R2, Maisha, China) according to the manufacturer's instructions.

### 2.18 Reverse Transcription‐Quantitative Polymerase Chain Reaction

Total RNA from primary neuron lysates was extracted with TRIzol reagent following the manufacturer's protocol (TrizolTM Reagent, Thermo Fisher Scientific, USA). The extracted RNA was reverse‐transcribed to cDNA using the TransScript One‐Step gDNA Removal and cDNA Synthesis SuperMix kit (AQ601‐04, TransGen Biotech, China). Relative mRNA levels were detected using SYBR Green Supermix (11201ES08, Yeasen Biotech Co. Ltd., China) on the LightCycler 480 (Roche, USA). The β‐actin was used as the internal control. The sequences of the primers used for qPCR are listed in Table S1.

### 2.19 Statistical Analysis

All data were presented as the mean ± SEM and analyzed using GraphPad Prism (version 8). Normality was assessed by applying Shapiro–Wilk tests, and data sets that failed these tests were analyzed nonparametrically. Subsequently, comparisons between two groups were made using two‐tailed Student's t tests, while comparisons among multiple groups were conducted using one‐way ANOVA followed by Tukey's post hoc tests, and *p* < 0.05 was considered statistically significant.

## 3 Results

### 3.1 Meth Exposure Contributed to Mitophagy and Necroptosis in Primary Neurons

**FIGURE 1:**
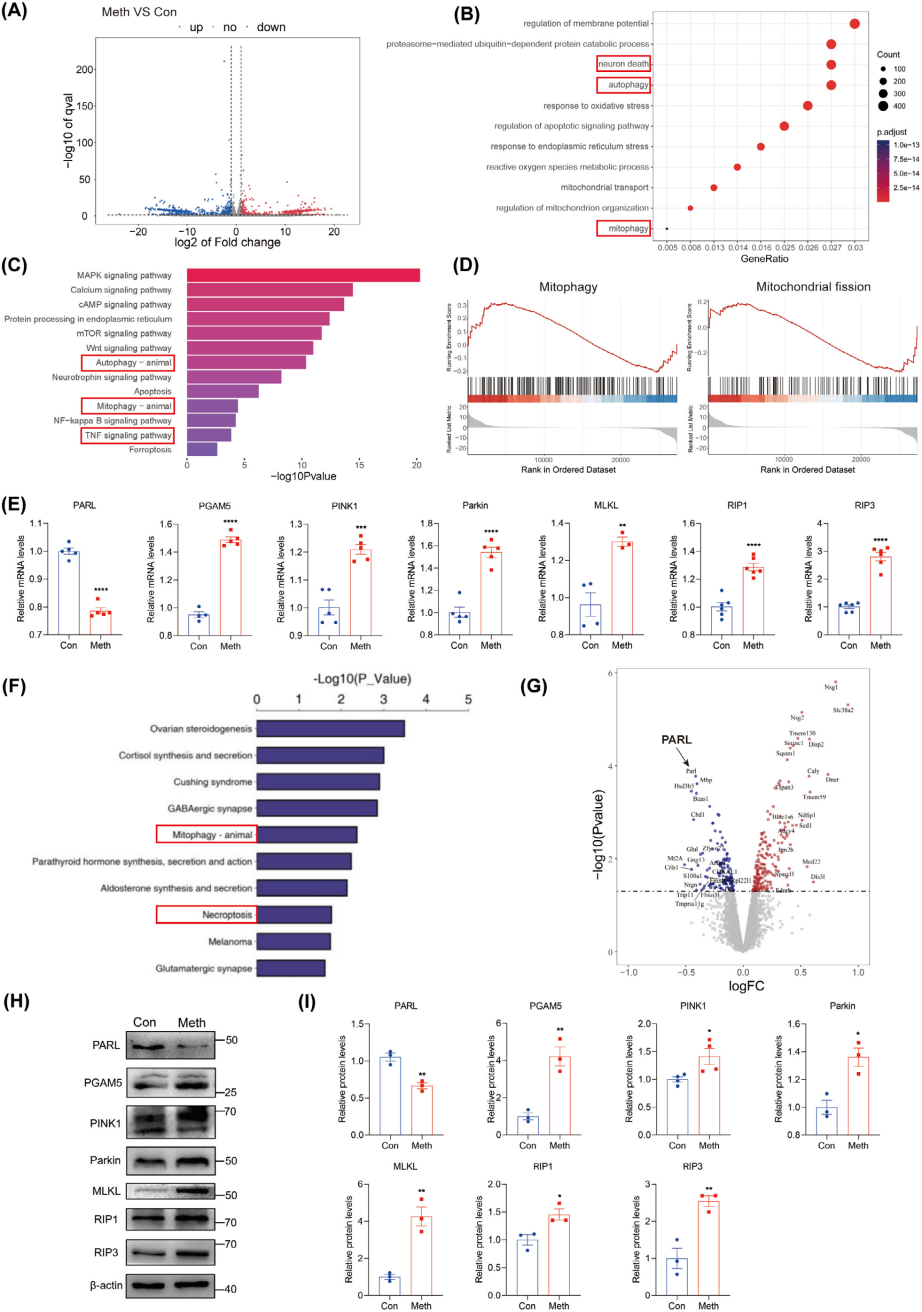
Meth exposure causes mitophagy and necroptosis in primary neurons. (A) Volcano plots of differential gene expression analysis. (B) GO pathway enrichment of differential gene expression analysis. (C) KEGG pathway enrichment of differential gene expression analysis. (D) Gene set enrichment analysis (GSEA) of mitophagy‐related and mitochondrial fission‐related RNA‐seq data. (E) PARL, PGAM5, PINK1, Parkin, MLKL, RIP1, and RIP3 mRNA levels in neurons. (F) KEGG pathway enrichment of differential protein expression analysis (Top 10). (G) Volca no plot of differential protein expression analysis. (H) PARL, PGAM5, PINK1, Parkin, MLKL, RIP1, and RIP3 protein levels. (I) Statistical results of the relative protein levels of PARL, PGAM5, PINK1, Parkin, MLKL, RIP1, and RIP3. Data are shown as the mean ± SEM. Student's t‐test was used to measure the significance between two groups. *p < 0.05, **p < 0.01, ***p < 0.001, and ****p < 0.0001 versus Con group.

Previous studies have found that binge administration of Meth ranging from 250 mg to 1 g results in brain concentration levels of Meth between 164 and 776 μM [35]. Thereafter, a dose of 900 μM Meth was used in the subsequent experiments were based on our previous studies [20, 21]. The transcriptome results identified 184 upregulated genes and 301 downregulated genes in primary neurons after Meth exposure (Figure 1A). GO enrichment analysis revealed these differentially expressed genes (DEGs) mainly participate in neuron death, autophagy, and mitophagy pathways (Figure 1B). Similarly, KEGG enrichment analysis showed substantial changes in autophagy, mitophagy, and TNF signaling pathways (Figure 1C). The importance of mitophagy and mitochondrial fission was further confirmed by GSEA analysis (Figure 1D). Mitophagy‐related genes PARL, PGAM5, PINK1, and Parkin, as well as necroptosis‐related genes MLKL, RIP1, and RIP3, all significantly increased in primary neurons after Meth treatment (Figure 1E). Besides, the proteomics analysis revealed 184 upregulated proteins and 121 downregulated proteins in the hippocampus tissue after Meth treatment. Of note, the mitophagy and necroptosis signaling pathways were obviously activated after Meth exposure (Figure 1F). Overall, PARL was one of the most significantly decreased proteins after Meth treatment (Figure 1G), which was validated at the gene and protein levels in primary neurons. In parallel, the protein levels of PGAM5, PINK1, Parkin, and the expression of MLKL, RIP1, and RIP3 in primary neurons were examined as well. Compared with the control group, these proteins were obviously increased after Meth exposure (Figure 1H,I), implying that mitophagy and necroptosis are involved in Meth‐induced neurotoxicity.

### 3.2 PARL Reversed the Meth‐Induced Neuronal Necroptosis in Primary Neurons

**FIGURE 2:**
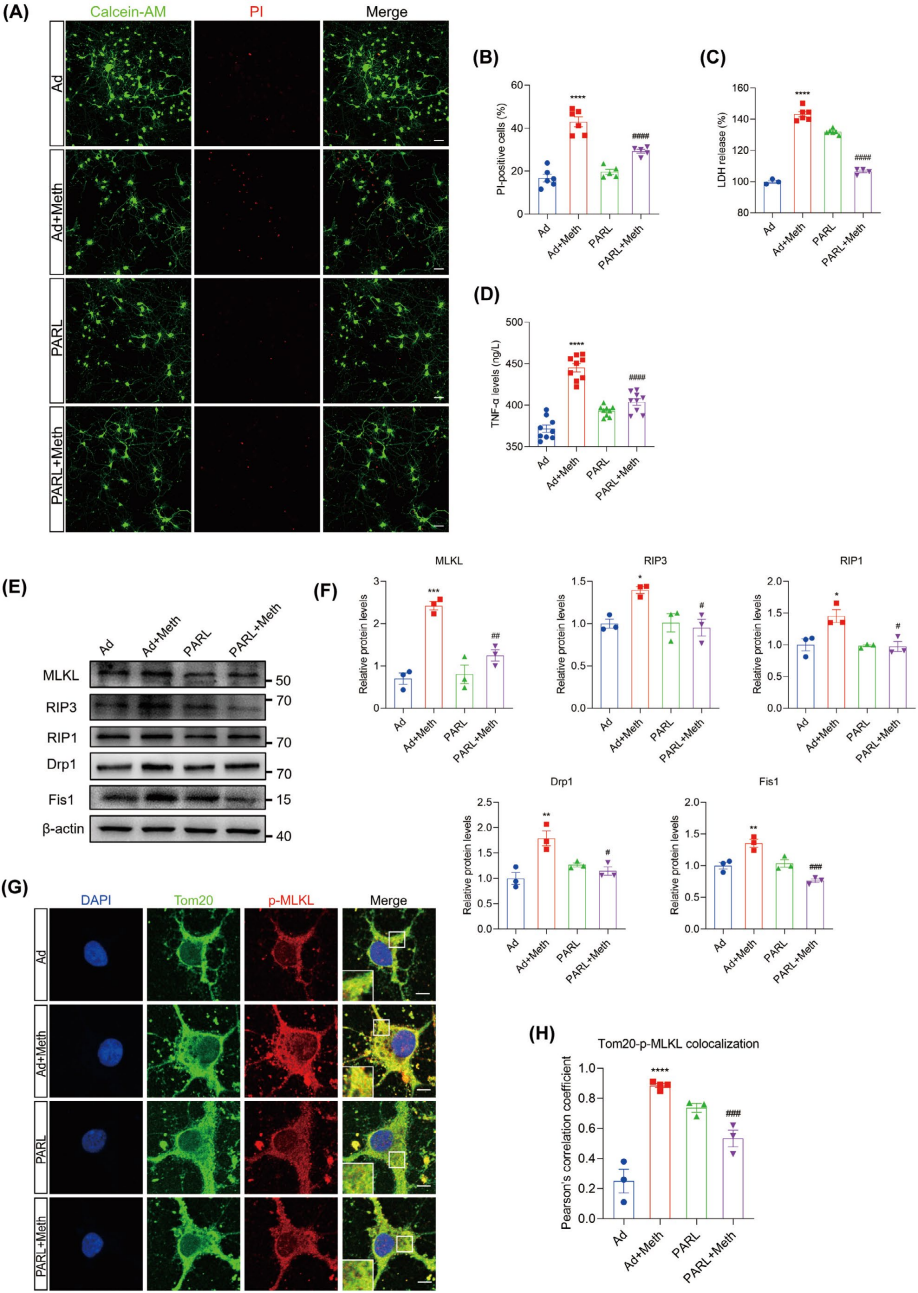
PARL overexpression reversed the Meth‐induced neuronal necroptosis. (A) The Calcein‐AM/PI double staining in control and PARL overexpression neurons after Meth treatment. Scale bar = 50 μm. (B) Quantitative analysis of the proportions of dead cells in neurons. (C) LDH levels in different groups. (D) The level of TNF‐α in the cell culture supernatants detected by ELISA. (E) MLKL, RIP3, RIP1, Drp1, and Fis1 protein expression. (F) Statistical results of the relative levels of MLKL, RIP3, RIP1, Drp1, and Fis1 proteins. (G) Tom20 (green) and p‐MLKL (red) co‐staining. Scale bar = 5 μm. (H) Quantitative analysis of colocalization of p‐MLKL and Tom20. Data are shown as the mean ± SEM. One‐way ANOVA was used to determine the statistical significance between multiple groups. *p < 0.05, **p < 0.01, ***p < 0.001, and ****p < 0.0001 versus Ad group; ^#^ *p* < 0.05, ^##^ *p* < 0.01, ^###^ *p* < 0.001, and ^####^ *p* < 0.0001 for the PARL + Meth group versus Ad + Meth group.

Since the critical role of PARL in PGAM5 modulation and the dramatic decline in PARL levels mediated by Meth, we investigated whether neuronal necroptosis can be rescued by PARL overexpression. Manifestly, as the Calcein‐AM/PI double staining displayed, PARL overexpression dramatically attenuated the Meth‐induced neuronal death (in red) (Figure 2A,B) and alleviated the membrane injury and TNF‐α excretion, since the marked decrease in LDH and TNF‐α levels in the cell supernatant (Figure 2C,D). The necrosome plays a critical role in necroptosis; therefore, it is reasonable to examine the necrosome formation. The colocalization of RIP1, RIP3, and MLKL was substantially increased in neurons subjected to Meth, an effect that could be markedly reversed by PARL overexpression (Figure S1G–J). Moreover, the RIP1, RIP3, and MLKL at gene and protein levels were significantly enhanced in the Meth‐treated group; after PARL overexpression, the increased levels of RIP1, RIP3, and MLKL were strikingly ameliorated (Figure S1A–F and Figure 2E,F). The activated MLKL (p‐MLKL) plays a vital role as it shuttles from the cytoplasm to the membrane, leading to membrane rupture. Intriguingly, p‐MLKL notably colocalized with the mitochondrial membrane marker Tom20 after Meth treatment, and the phenomenon was reversed by PARL overexpression (Figure 2G,H). Hence, these results suggest that Meth exposure promotes necrosome generation and activates the MLKL, leading to mitochondrial membrane permeabilization, whereas PARL plays a critical role in alleviating these adverse outcomes.

### 3.3 PARL Attenuated Neuronal Mitochondrial Dysfunction Induced by Meth

**FIGURE 3:**
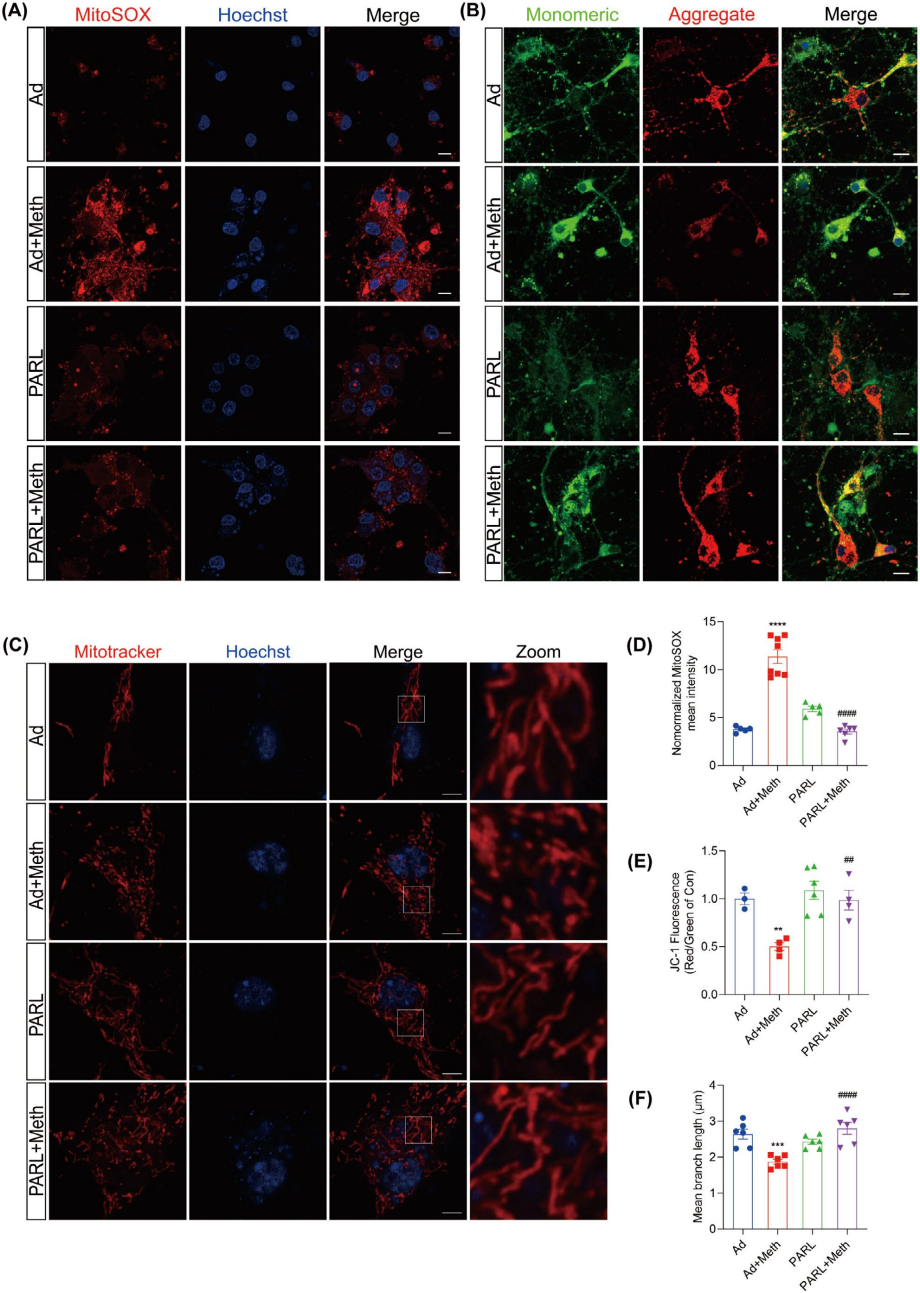
PARL overexpression attenuated neuronal mitochondrial dysfunction induced by Meth. (A, D) Mitochondrial ROS generation in neurons and quantification showing the relative Mitosox fluorescence intensity. Scale bar = 10 μm. (B, E) MMP changes in neurons and quantification assessed by JC‐1 staining. Scale bar = 20 μm. (C, F) MitoTracker staining and quantification of mean branch length showing mitochondrial morphology in neurons. Scale bar = 5 μm. **p < 0.01, ***p < 0.001, and ****p < 0.0001 versus Ad group; ^##^ *p* < 0.01 and ^####^ *p* < 0.0001 for the PARL + Meth group versus Ad + Meth group.

Given the critical role of ROS in triggering necrosome formation [9], Mitosox, a probe specific for mitochondrial ROS, was employed. As expected, Meth treatment resulted in excessive mitochondrial ROS production; however, this effect could be substantially impeded by PARL overexpression, suggesting the beneficial roles of PARL in mitochondrial ROS inhibition (Figure 3A,D). Since mtROS is closely associated with both mitochondrial depolarization and fragmentation [36], the alterations in mitochondrial morphology by Mitotracker were examined. It showed that Meth significantly shortened the mitochondria, leading to mitochondrial fragmentation, whereas PARL overexpression dramatically suppressed this process and restored mitochondrial morphology (Figure 3C,F). To address the phenomenon of the Meth‐induced alterations of mitochondrial morphology, the expression of mitochondrial fission proteins Drp1 and Fis1 at gene and protein levels was explored (Figure S1E,F; Figure 2E,F); in line with the phenotype, Drp1 and Fis1 levels were obviously increased, an effect that was ameliorated by PARL overexpression. Mitochondrial fragmentation was reported to affect mitochondrial membrane potential (MMP); thus, JC‐1, a probe for MMP detection, was applied. As shown in Figure 3B,E, compared with the control neurons (normal MMP with red fluorescence), the Meth‐treated neurons displayed significantly decreased red fluorescence and reciprocally enhanced green fluorescence, and the phenomenon could be reversed by PARL overexpression. Taken together, PARL overexpression prevents the Meth‐induced mitochondrial injury by decreasing mtROS generation, mitochondrial fragmentation, and impeding mitochondrial membrane depolarization.

### 3.4 PARL Regulates Differential Cleavage of PINK1 and PGAM5 After Meth Exposure

**FIGURE 4:**
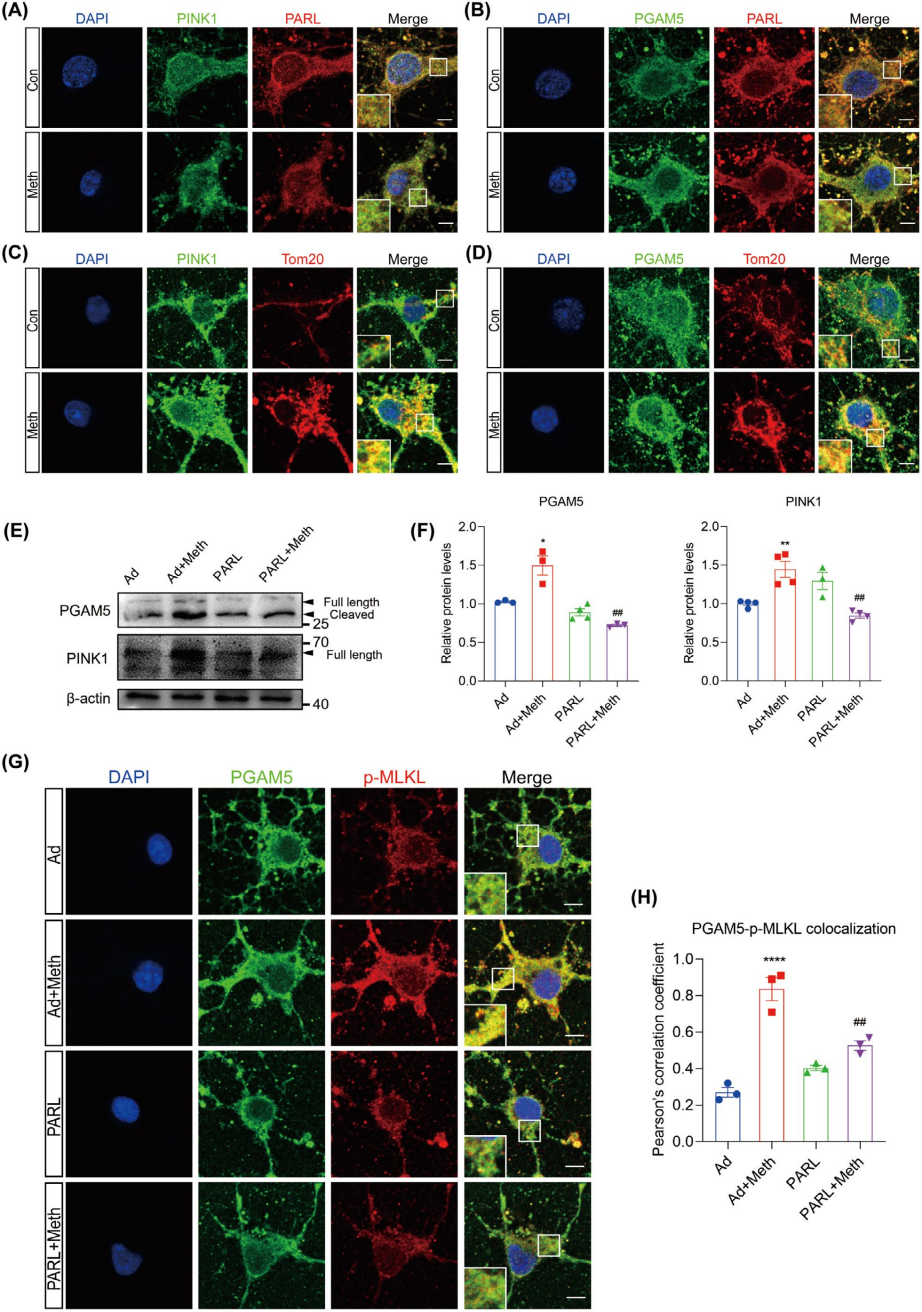
PARL regulates PINK1 and PGAM5 cleavage after Meth exposure. (A) PINK1 (green) and PARL (red) costaining. Scale bar = 5 μm. (B) PGAM5 (green) and PARL (red) costaining. Scale bar = 5 μm. (C) PINK1 (green) and Tom20 (red) costaining. Scale bar = 5 μm. (D) PGAM5 (green) and Tom20 (red) costaining. Scale bar = 5 μm. (E) Detection of PGAM5 and PINK1 protein expression by Western blot. (F) Statistical results of the relative expression levels of PGAM5 and PINK1 proteins. (G) PGAM5 (green) and p‐MLKL (red) costaining. Scale bar = 5 μm. (H) Quantitative analysis of colocalization of PGAM5 and p‐MLKL. *p < 0.05, **p < 0.01, and ****p < 0.0001 versus Ad group; ^##^ *p* < 0.01 for the PARL + Meth group versus Ad + Meth group.

The cleavage of PINK1 and PGAM5 by PARL is dependent on the status of mitochondria [29]. To decipher the cleavage properties modulated by PARL, the colocalization among PARL, PINK1, and PGAM5 was examined after Meth treatment. As shown in Figure 4A,B; Figure S3A,B, the colocalization between PARL and PINK1 was dramatically decreased and reciprocally markedly increased between PARL and PGAM5 in the Meth group. Besides, the full‐length PINK1, full‐length PGAM5, and cleaved PGAM5 protein levels were significantly increased, mediated by Meth, an effect that was substantially rescued by PARL overexpression (Figure 4E,F). Intriguingly, an increased colocalization of PINK1 and PGAM5 with Tom20 was observed after Meth exposure (Figure 4C,D; Figure S3C,D), suggesting a plausible connection between mitochondrial dysfunction and neuronal injury. Since PGAM5 acts as an exacerbating factor for mitochondrial fission and neuronal death, it is important to determine whether the PARL‐mediated cleavage of PGAM5 plays a critical role in the regulation of necroptosis. As shown in Figure 4G,H, PGAM5 exhibited a significant increase in colocalization with p‐MLKL following Meth exposure, and this effect was strikingly ameliorated by PARL overexpression. These results indicate that PARL overexpression ameliorates the Meth‐induced PGAM5 cleavage and PGAM5‐p‐MLKL complex formation in the mitochondria outer membrane.

### 3.5 PARL Ameliorated Excessive Mitophagy and Autophagic Flux Defects in Primary Neurons

**FIGURE 5:**
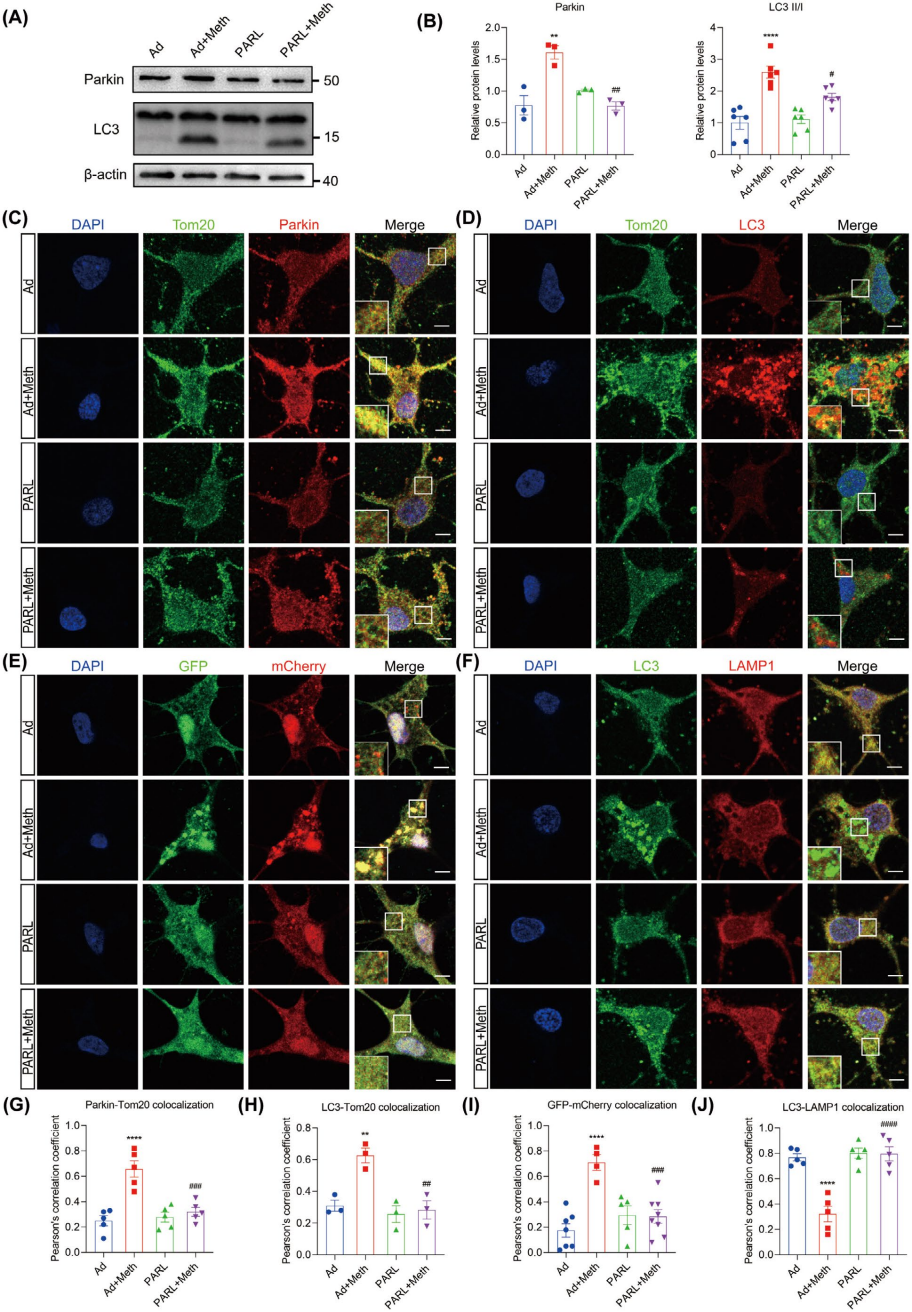
PARL overexpression rescued excessive mitophagy and defective autophagic flux in primary neurons. (A) Detection of Parkin and LC3 protein expression by Western blot. (B) Statistical results of the relative expression levels of Parkin and LC3II/I proteins. (C) Tom20 (green) and Parkin (red) costaining. Scale bar = 5 μm. (D) Tom20 (green) and LC3 (red) costaining. Scale bar = 5 μm. (E, I) Fluorescence analysis of neurons transfected with mCherry‐GFP‐LC3 adenovirus. The yellow puncta indicate autophagosomes, and the free red puncta indicate autolysosomes. Scale bar = 5 μm. (F) LC3 (green) and LAMP1 (red) costaining. Scale bar = 5 μm. (G) Quantitative analysis of colocalization of Parkin and Tom20. (H) Quantitative analysis of colocalization of LC3 and Tom20. (J) Quantitative analysis of colocalization of LC3 and LAMP1. **p < 0.01 and ****p < 0.0001 versus Ad group; ^#^ *p* < 0.05, ^##^ *p* < 0.01, ^###^ *p* < 0.001, ^####^ *p* < 0.0001 for the PARL + Meth group versus Ad + Meth group.

Since both PINK1 and cleaved PGAM5 stimulate the process of mitophagy, we then sought to investigate whether PARL overexpression ameliorates the excessive mitophagy induced by Meth. The immunofluorescence (IF) staining showed a significant increase in the colocalization of Parkin and Tom20 in primary neurons after Meth exposure. Moreover, LC3, a marker protein of the autophagosome, was strongly colocalized with Tom20, indicating excessive mitophagy. Notably, these effects were strikingly reversed by PARL overexpression (Figure 5C,D,G,H). Consistent with this phenomenon, the protein levels of Parkin, LC3II, and p62 markedly increased after Meth challenge, along with enhanced conversion from LC3I to LC3II, and these effects can be strikingly reversed by PARL overexpression (Figure 5A,B; Figure S3E,F). To further verify the autophagic defects, an mCherry‐GFP‐LC3‐expressing adenovirus commonly used to examine autophagic flux was applied. It showed that Meth treatment exhibited a massive accumulation of colocalized mCherry‐GFP yellow puncta in primary neurons, indicating defective autophagic flux. Noteworthily, PARL overexpression obviously improved the autophagic flux, evidenced by the decreased numbers of yellow puncta (Figure 5E,I). Consistently, Meth also reduced the colocalization of LC3 and LAMP1, confirming the blockage of autophagosome‐lysosome fusion, and this process, similarly, can be strikingly reversed by PARL overexpression (Figure 5F,J).

### 3.6 Knockdown of PGAM5 Alleviated Necroptosis and Mitochondrial Dysfunction in Primary Neurons

**FIGURE 6:**
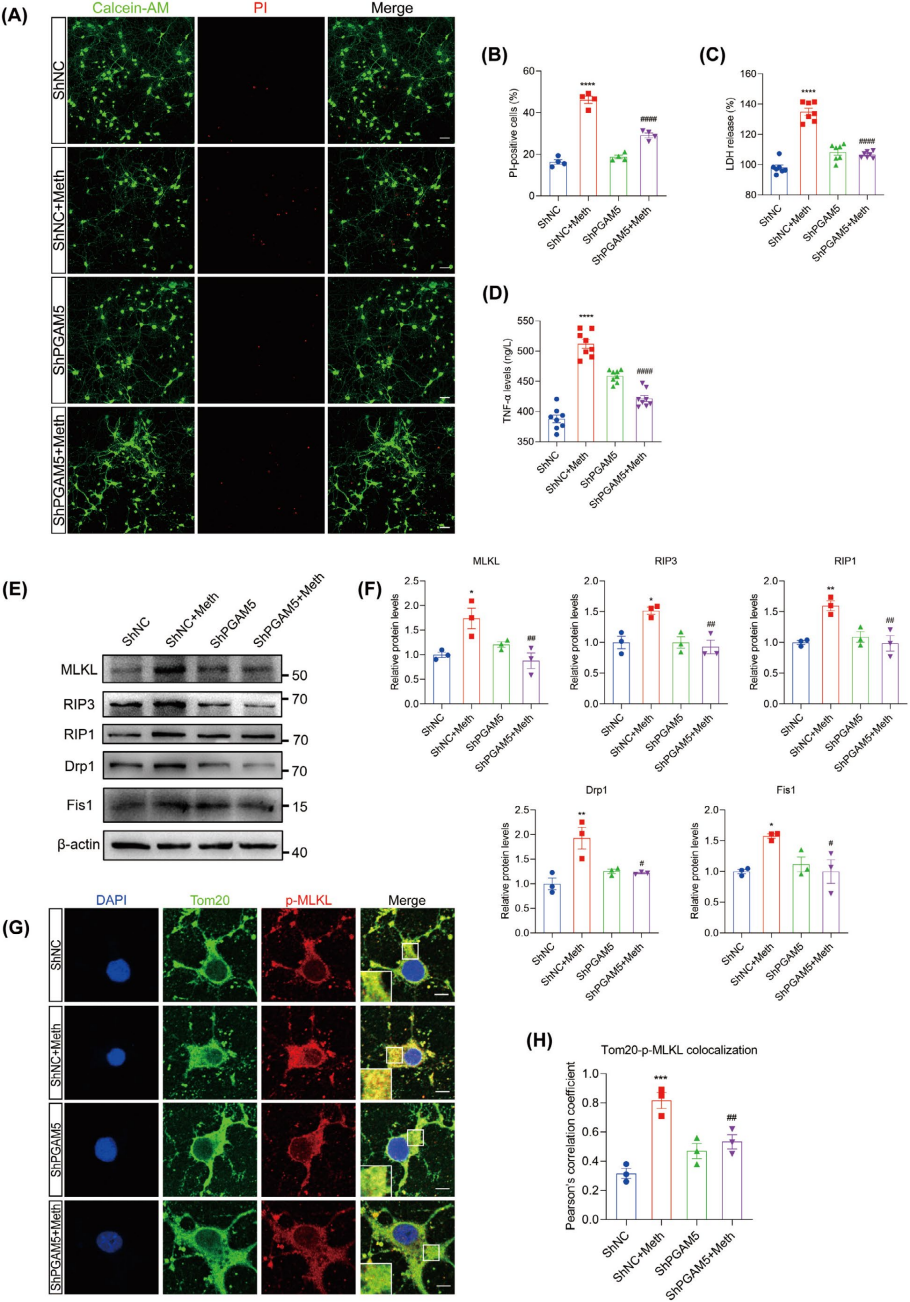
Knockdown of PGAM5 alleviated neuronal necroptosis in primary neurons. (A) The Calcein‐AM/PI double staining in control and PGAM5 downregulation neurons after Meth treatment. Scale bar = 50 μm. (B) Quantitative analysis of the proportions of dead cells in neurons. (C) The LDH release level was measured in different groups. (D) The expression level of TNF‐α in the cell culture supernatants was analyzed by ELISA. (E) Detection of MLKL, RIP3, RIP1, Drp1, and Fis1 protein expression by Western blot. (F) Statistical results of the relative expression levels of MLKL, RIP3, RIP1, Drp1, and Fis1 proteins. (G) Tom20 (green) and p‐MLKL (red) costaining. Scale bar = 5 μm. (H) Quantitative analysis of colocalization of p‐MLKL and Tom20. *p < 0.05, **p < 0.01, ***p < 0.001, and ****p < 0.0001 versus ShNC group; ^#^ *p* < 0.05, ^##^ *p* < 0.01, and ^####^ *p* < 0.0001 for the ShPGAM5 + Meth group versus ShNC + Meth group.

**FIGURE 7:**
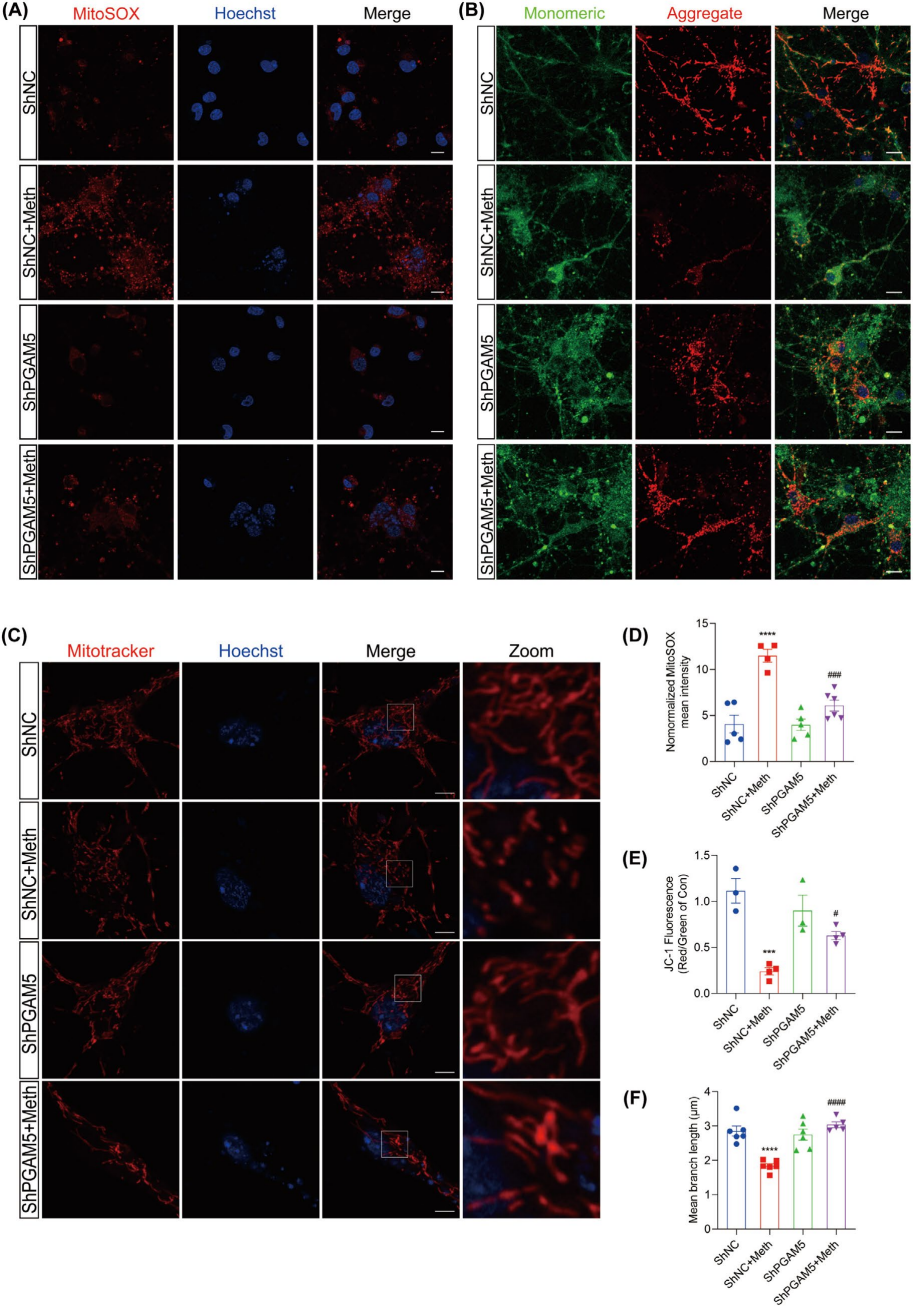
Knockdown of PGAM5 alleviated mitochondrial dysfunction in primary neurons. (A, D) Mitochondrial ROS generation in neurons and quantification showing the relative Mitosox fluorescence intensity. Scale bar = 10 μm. (B, E) Mitochondrial membrane potential changes in neurons and quantification assessed by JC‐1 staining. Scale bar = 20 μm. (C, F) MitoTracker staining and quantification of mean branch length showing mitochondrial morphology in neurons. **p < 0.01, ***p < 0.001, and ****p < 0.0001 versus ShNC group; ^#^ *p* < 0.05, ^###^ *p* < 0.001, and ^####^ *p* < 0.0001 for the ShPGAM5 + Meth group versus ShNC + Meth group.

PGAM5 was demonstrated to be the converging signal of multiple necroptosis pathways [32], and it is logical to investigate whether PGAM5 knockdown alleviated the Meth‐induced mitochondrial injury and neuronal necroptosis. As expected, the knockdown of PGAM5 obviously decreased PI‐positive cells, LDH, and TNF‐α release (Figure 6A–D). Moreover, PGAM5 knockdown significantly impeded the Meth‐induced necrosome formation, MLKL, RIP3, and RIP1 expression (Figure 6E,F; Figure S2B–D,G–J) and retarded MLKL transferring to the mitochondrial membrane (Figure 6G,H), thereby attenuating mtROS generation, MMP decrease, and mitochondrial fragmentation (Figure 7A–F). For the mitochondrial dynamics analysis, Meth obviously increased Drp1 and Fis1 at the mRNA and protein levels (Figure S2E,F; Figure 6E,F), whereas PGAM5 knockdown obviously reversed this effect. These data underscored the key roles of PGAM5 in Meth‐induced mitochondrial dysfunction and neuronal necroptosis.

### 3.7 PARL Overexpression and PGAM5 Knockdown Ameliorated Meth‐Induced Neuronal Necroptosis and Cognitive Defects in Mice

**FIGURE 8:**
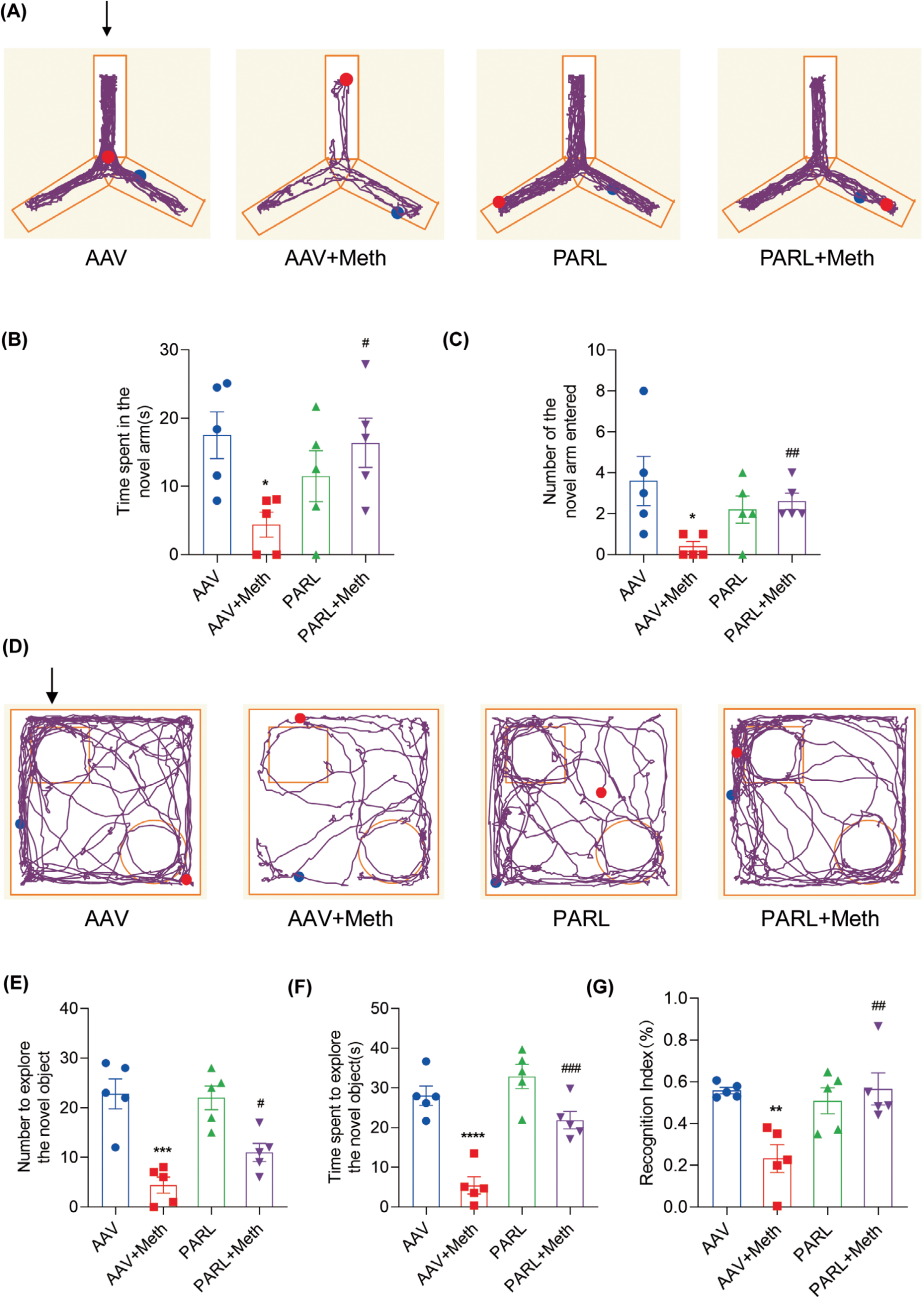
PARL overexpression improved cognitive learning after Meth exposure. (A) Trajectory of mice in the Y‐maze experiment. The arrow indicates the novel arm. (B) Time spent in the novel arm within 5 min. (C) Number of times the mice entered the novel arm within 5 min. (D) Trajectory of mice in the novel object recognition test (NOR). The arrow indicates the novel object. (E) Number to explore the novel object within 5 min. (F) Time spent to explore the novel object within 5 min. (G) The recognition index of mice was calculated. *p < 0.05, **p < 0.01, ***p < 0.001, and ****p < 0.0001 versus AAV group; ^#^ *p* < 0.05, ^##^ *p* < 0.01, and ^###^ *p* < 0.001 for the PARL + Meth group versus AAV + Meth group.

**FIGURE 9:**
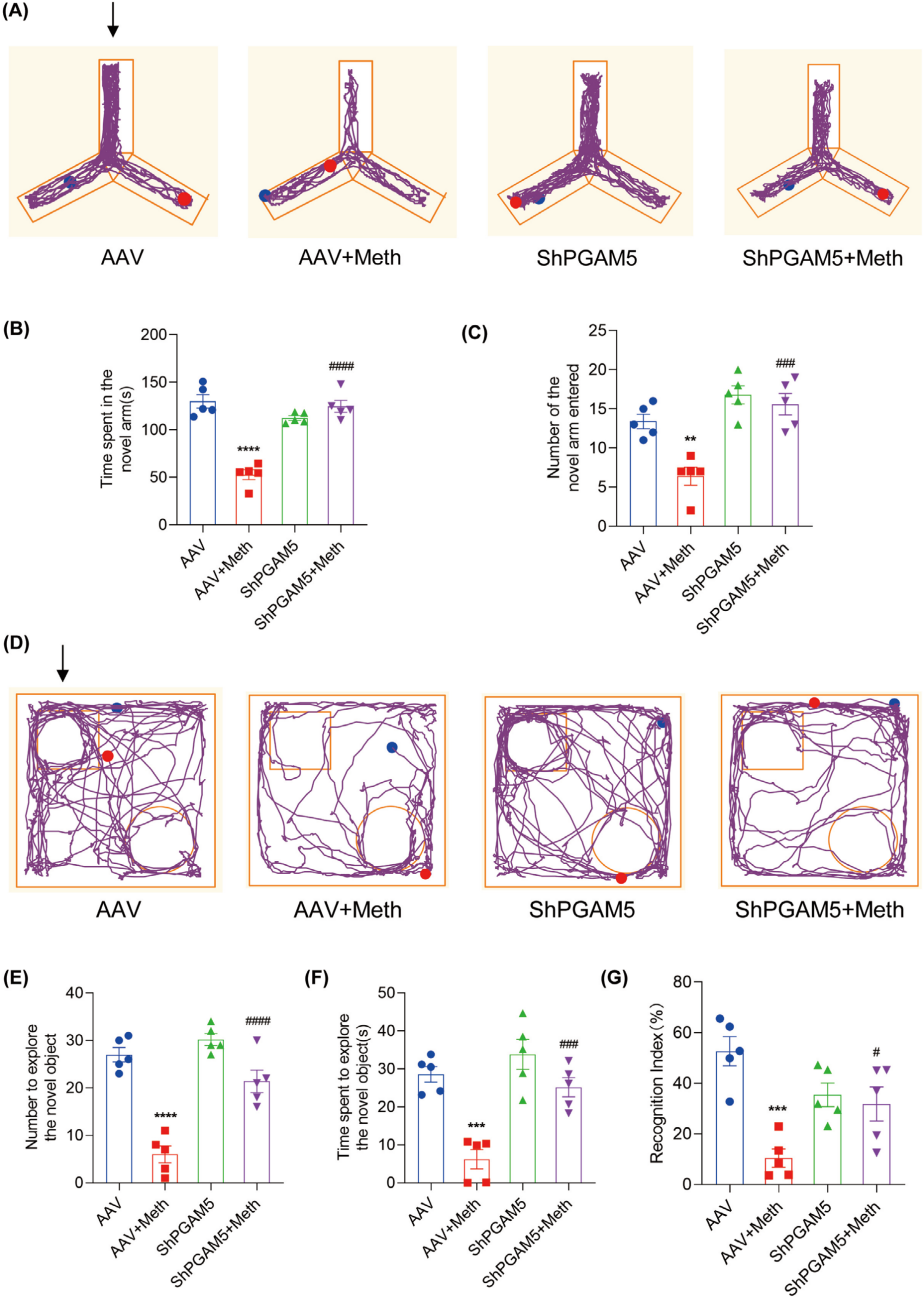
PGAM5 knockdown improved cognitive learning after Meth exposure. (A) Trajectory of mice in the Y‐maze experiment. The arrow indicates the novel arm. (B) Time spent in the novel arm within 5 min. (C) Number of times the mice entered the novel arm within 5 min. (D) Trajectory of mice in the novel object recognition test (NOR). The arrow indicates the novel object. (E) Number to explore the novel object within 5 min. (F) Time spent exploring the novel object within 5 min. (G) The recognition index of mice was calculated. **p < 0.01, ***p < 0.001, and ****p < 0.0001 versus AAV group; ^#^ *p* < 0.05, ^###^ *p* < 0.001, and ^####^ *p* < 0.0001 for the ShPGAM5 + Meth group versus AAV + Meth group.

**FIGURE 10:**
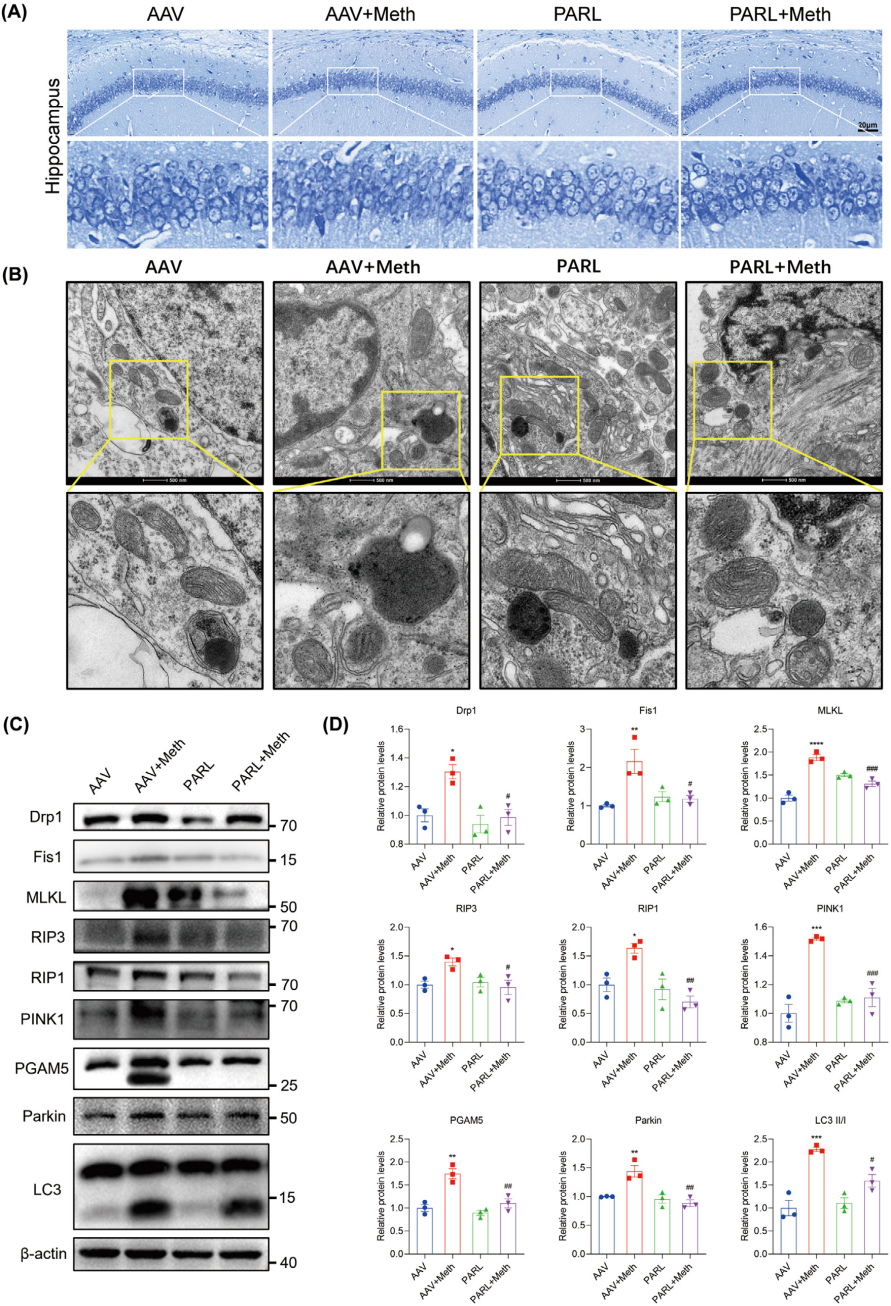
PARL overexpression ameliorated neuronal loss after Meth exposure. (A) PARL overexpression significantly increased the number of Nissl‐positive cells in the hippocampus after Meth exposure. (B) TEM images showing mitochondrial morphology and autophagosome containing unhealthy mitochondria in the hippocampus. (C) Detection of Drp1, Fis1, MLKL, RIP3, RIP1, PINK1, PGAM5, Parkin, and LC3 protein expression by Western blot. (D) Statistical results of the relative expression levels of Drp1, Fis1, MLKL, RIP3, RIP1, PINK1, PGAM5, Parkin, and LC3 proteins. *p < 0.05, **p < 0.01, ***p < 0.001, and ****p < 0.0001 versus AAV group; ^#^ *p* < 0.05, ^##^ *p* < 0.01, and ^###^ *p* < 0.001 for the PARL + Meth group versus AAV + Meth group.

**FIGURE 11:**
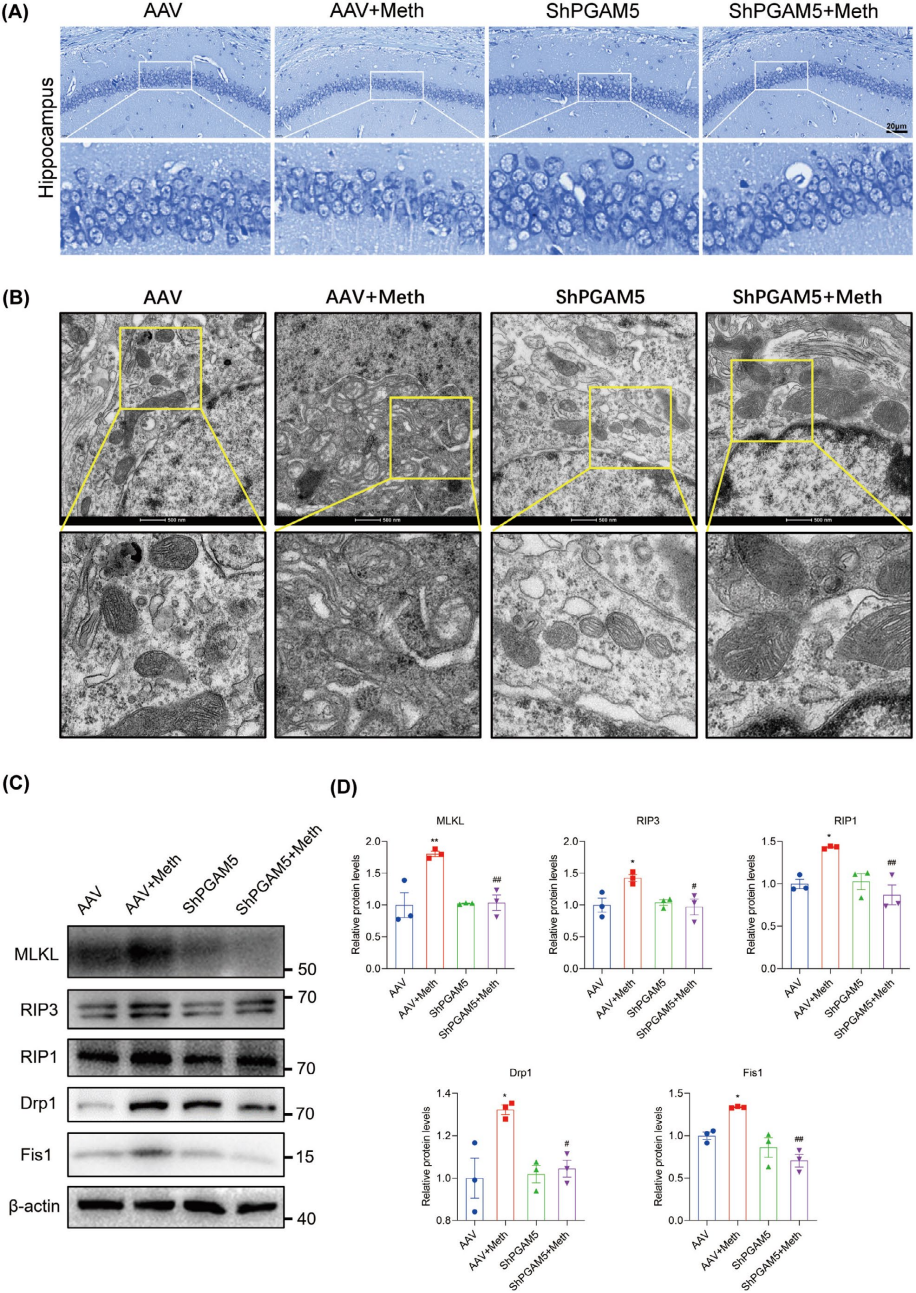
PGAM5 knockdown ameliorated neuronal loss after Meth exposure. (A) PGAM5 downregulation significantly increased the number of Nissl‐positive cells in the hippocampus after Meth exposure. (B) TEM images showing mitochondrial morphology and autophagosome containing unhealthy mitochondria in the hippocampus. (C) Detection of MLKL, RIP3, RIP1, Drp1, and Fis1 protein expression by Western blot. (D) Statistical results of the relative expression levels of MLKL, RIP3, RIP1, Drp1, and Fis1 proteins. *p < 0.05 and ^**^ *p* < 0.01, versus AAV group; ^#^ *p* < 0.05 and ^##^ *p* < 0.01 for the ShPGAM5 + Meth group versus AAV + Meth group.

Having determined the crucial roles of the PARL‐PGAM5 signaling axis in Meth‐induced neuronal necroptosis in vitro, we then explored whether these effects can be validated in the in vivo study. Therefore, the PARL‐AAV or ShPGAM5‐AAV was stereotaxically injected into the CA1 and CA3 regions of the mice hippocampus (Figure S3K–N). After 3 weeks, mice were injected with binge doses of Meth (4 × 10 mg/kg, 2–3 h intervals) or saline (Figure S3O). The Y‐maze test revealed that there was a significant decrease in the number of entries and duration of time spent in the novel arm induced by Meth compared to mice with PARL overexpression or PGAM5 knockdown (Figures 8A–C and 9A–C). Additionally, the new object recognition test showed that Meth exposure markedly reduced the time spent and number of explorations of the novel object, with a lesser recognition index. As expected, both PARL overexpression and PGAM5 knockdown could rescue the Meth‐induced cognitive impairments (Figures 8D–G and 9D–G). Pathologically, the neuronal cells were loosely arranged and decreased, and Nissl bodies were lightly stained or even dissolved compared with PARL overexpression and PGAM5 knockdown cells (Figures 10A and 11A; Figure S3I,J), suggesting that PARL overexpression or PGAM5 knockdown exerts a salutary effect on cognitive and spatial learning against Meth‐induced memory impairments. Meanwhile, TEM was used to evaluate the formation and accumulation of mitophagosomes in the mouse hippocampus. As shown in Figures 10B and 11B, mitochondria in Meth‐treated neurons displayed fragmentation with reduced or no cristae structures and ruptured membranes, which were engulfed by the accumulated autophagosomes, while PARL overexpression or PGAM5 knockdown remarkably restored the Meth‐induced subcellular structure impairments. On the basis of the aforementioned results, the expression levels of mitophagy‐related proteins, including PINK1, PGAM5, Parkin, LC3II/I, and p62 in the hippocampus were examined. In line with the results represented in TEM, these proteins were increased in the Meth group, an effect that was dramatically reduced by PARL overexpression (Figure 10C,D; Figure S3G,H). Additionally, mitochondrial dynamics play a key role in the response of cells to oxidative stress, and mitochondrial fission is necessary to trigger mitophagy. Unsurprisingly, PARL overexpression or PGAM5 knockdown significantly suppressed the Meth‐induced increase of mitochondrial fission proteins, Drp1 and Fis1. Furthermore, the necroptosis proteins, including MLKL, RIP1, and RIP3, were significantly inhibited as well (Figures 10C,D and 11C,D). Taken together, these findings indicate that targeting PARL‐PGAM5 signaling plays pivotal salutary effects against Meth‐mediated brain injury.

## 4 Discussion

In the current study, we uncover a mechanism of Meth‐induced neuronal necroptosis involving mitochondrial dysfunction, which may be partially ascribed to mitophagy‐associated necroptosis. This study, to our knowledge, is the first time to reveal the PARL‐mediated aberrant cleavage of PINK1 and PGAM5, contributing to necrosome formation, mitochondrial injury, and excessive mitophagy, which finalize neuronal necroptosis.

Necroptosis is characterized by the rapid rupture of the cell membrane, swelling of organelles, and outflow of intracellular contents [6, 7, 8]. Meth treatment significantly promoted neuronal death partially by disrupting membrane integrity, evidenced by an increased PI positive rate and enhanced LDH excretion. The necroptotic pathway involves TNF‐α‐regulated signaling through RIP1, RIP3, and MLKL [37]. In the cytoplasm, RIP1 binds to RIP3, forming a complex known as the necrosome, which then interacts with MLKL. Accumulating evidence has recently addressed the association between necroptosis and Meth‐induced neurotoxicity. In both humans and mice, Meth exposure exhibited a pronounced increase of RIP3 and MLKL in the striatum [13, 38]. Besides, both the RIP1 inhibitor Nec‐1 and the RIP3 inhibitor GSK872 were reported to effectively mitigate neurotoxicity induced by Meth [14, 39]. In the present study, with the binge doses (4 × 10 mg/kg, 2–3 h intervals) of Meth, the mice exhibited poor cognitive performance, ascribed to the increased damaged neurons, evidenced by less Nissl body staining. At the molecular level, the increased TNF‐α expression and colocalization of RIP3 with RIP1 or MLKL were observed. Therefore, targeting necroptosis‐related proteins could be considered a promising therapeutic strategy for Meth‐induced acute brain injury.

Plasma membrane pore formation is a key feature of necroptosis [40, 41], characterized as the translocation of p‐MLKL to the cell membrane [11, 42]. Unexpectedly, in the current study, p‐MLKL was also proved to translocate to the outer mitochondrial membrane, which may trigger MMP decrease. In fact, the role of mitochondria in Meth‐induced neurotoxicity has been emphasized recently, and the underlying mechanisms may include oxidative stress, inflammatory, and mitochondrial dysfunction [43, 44, 45]. Researchers have observed dose‐dependent mitochondrial structural alterations after Meth exposure, accompanied by ROS production, misfolded protein accumulation, and decreased cell viability, thereby triggering the mitochondrial‐dependent death pathway [16, 46]. In the current study, we expanded the findings of the p‐MLKL‐mediated mitochondrial membrane damage by the evidence of massive overlapping of p‐MLKL and Tom20, and the resultant decreased MMP, increased mtROS, and mitochondrial fragmentation. Besides, the mitochondrial ultrastructure of the neurons in the Meth group displayed scattered, swollen mitochondria with abnormal cristae, along with vacuolization, swelling, and integrity loss, which strengthens the mitochondrial as the potential target of Meth. Understanding the crosslink between mitochondrial dysfunction and necroptosis may provide insights into the development of therapeutic strategies targeting Meth‐induced neuronal loss.

Mitophagy is an important mitochondrial quality control mechanism that eliminates damaged mitochondria. However, excessive mitophagy is linked to massive ROS generation [26, 47], since excessive clearance of mitochondria, which is deleterious in terms of normal cellular requirements, disturbs the intricate mitochondrial homeostasis [48]. Evidence has shown that autophagosomes containing damaged mitochondria are found in injured neurons, indicating excessive mitophagy in the early stages of injury [49, 50]. In line with the aforementioned phenotypes the TEM results showed massive autophagosome accumulation, containing fragmented mitochondria after Meth treatment. In fact, excessive accumulation of misfolded or impaired mitochondria in the autophagosomes has been shown to induce a stress response, activating programmed cell death via the mitochondrial cell death pathway, thereby inducing mitophagy‐dependent necroptosis [51, 52].

PGAM5 plays pivotal roles in mitophagy and necroptosis. It presents as two splice variants, the full‐length form and the cleaved form, and both isoforms function in the intrinsic necroptosis pathway [32]. Under stress conditions, PGAM5 serves as a binding target for RIP3, recruiting necrosomes to mitochondria and activating Drp1, facilitating mitochondrial fragmentation, an early and obligatory step for necroptosis [32]. On the contrary, pharmacological blockage of PGAM5 plays a protective effect on cerebral vessels and maintains blood–brain barrier function [53], by reducing neuroinflammation and suppressing oxidative stress [54, 55, 56]. In the current study, Meth exposure resulted in an increase of both full‐length and cleaved PGAM5, and after PGAM5 inhibition, the Meth‐induced mtROS, mitochondrial fragmentation, and mitochondrial depolarization were all obviously attenuated, underscoring the pleiotropic roles of PGAM5 in Meth‐induced mitochondrial fragment and membranal injury. Another interesting finding in the present study is the massive co‐staining of PAGM5 and p‐MLKL under Meth challenge, indicating the interaction between PGAM5 and p‐MLKL, which may contribute to pore formation on the mitochondrial membrane, leading to mitochondrial MMP decrease and excessive mitophagy. In congruence with the results in the in vitro study, the in vivo study revealed an increase in neuronal survival and improved cognitive behaviors following PGAM5 conditional knockdown in the hippocampus after Meth exposure, underlining the key roles of PGAM5 in Meth‐induced neuronal injury. Nonetheless, the full knowledge of how Meth‐mediated necroptosis through PGAM5 remains yet to be known.

PARL is located in the inner mitochondrial membrane and known as a critical regulator in the maintenance of mitochondrial structure and function [57]. Severe neurological alterations have also been observed in flies carrying mutations in PARL, accompanied by mitochondrial ultrastructural abnormalities that accumulate over time [58]. Besides, PARL mutation may partially contribute to the formation of Lewy bodies and is associated with the pathogenesis of Parkinson’ disease [59]. Intriguingly, PARL regulates differential cleavage of PINK1 and PGAM5. Specifically, in polarized mitochondria, PARL preferentially cleaves PINK1, and both substrates play vital roles in mitophagy [29]. After mitochondrial depolarization, MMP loss induces the blockage of the PINK1 cleavage and reciprocally enhanced PGAM5 cleavage mediated by PARL. In the present study, Meth exposure contributed to a pronounced reduction of PARL in neurons; moreover, the colocalization between PARL and PINK1 was dramatically decreased than that between PARL and PGAM5 in Meth group.

Furthermore, we found that full‐length form of PINK1 and full‐length and cleaved forms of PGAM5 protein expression were obviously increased concomitant with the accumulation of PINK1 and PGAM5 on the OMM. Studies have reported that full‐length PGAM5 stabilizes PINK1 to retain on the OMM. Since both full‐length and cleaved forms of PGAM5 play crucial roles in the regulation of necroptosis [29, 30, 31, 33], the downregulation of PARL following Meth treatment induced an increase in PGAM5 and PINK1 that resulted in excessive mitophagy and subsequent neuronal necroptosis. To further validate the causal relationship between PARL and mitophagy, PARL was upregulated both in vivo and in vitro studies. In concert with our assumption, PARL overexpression markedly ameliorated neuronal loss, mitochondrial injury, and cognitive impairments after Meth exposure; Besides, the Meth‐induced upregulation of PINK1, PGAM5, Parkin, MLKL, RIP1, and RIP3 expression at gene and protein levels was substantially ameliorated, strongly confirming the salutary effects of PARL on Meth‐induced neurotoxicity.

Another intriguing finding of the current study is that the overexpression of PARL facilitated the decreased cleaved PGAM5. Here, PARL ameliorated the Meth‐induced defects of autophagic flux, which may account for the decreased cleaved PGAM5. In our previous work, Meth treatment significantly activated factors that are beneficial to autophagic flux, as it increased lysosome number and upregulated LAMP1. Interestingly, Stx17, which plays a crucial role in autophagosome‐lysosome fusion, was significantly decreased [20, 21]. These findings suggested that the autophagosome‐lysosome fusion defects rather than lysosome dysfunction are involved in Meth‐induced neuronal damage. However, the reasons for the PARL‐enhanced autophagic flux are unknown, and further experiments are required to elucidate the underlying mechanisms. Thus, the PARL‐PGAM5 axis may be potential therapeutic targets to improve neuronal injury after Meth exposure. However, there are still several limitations in the current work. First, how PARL modulates autophagic flux and facilitates pathological protein degradation deserves further studies; Second, the direct evidence for p‐MLKL‐mediated alteration of mitochondrial membrane permeability remains enigmatic, and more studies are required to address the aforementioned questions.

**FIGURE 12:**
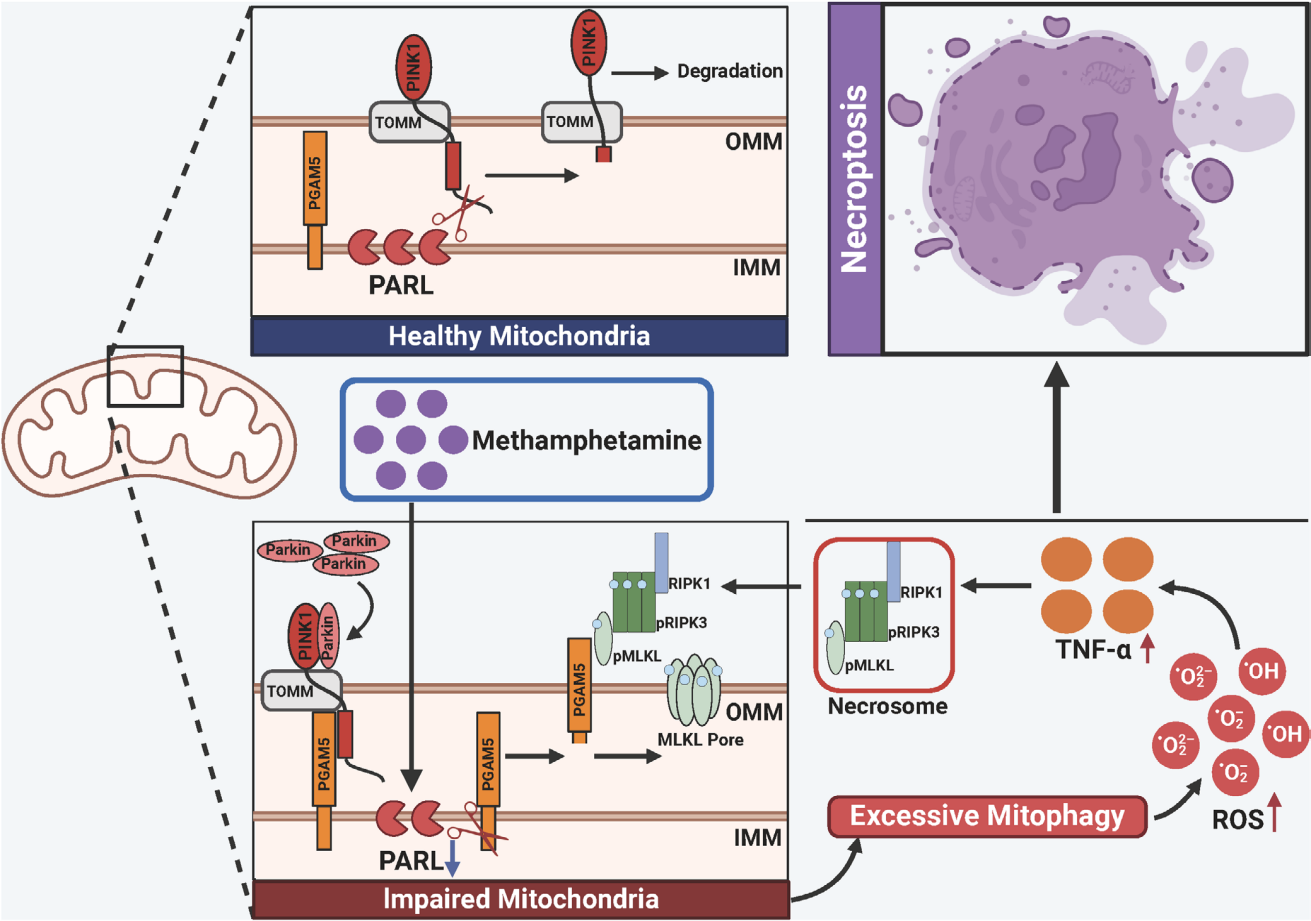
Schematic diagram of the effects of Meth on mitophagy and necroptosis in neurons. Low‐expression PARL induced by Meth regulated the differential cleavage of PINK1 and PGAM5, leading to the accumulation of PGAM5, which in turn recruited p‐MLKL on the mitochondrial membrane, forming “pores” that disrupted mitochondrial homeostasis, aggravating ROS production and TNF‐α release, ultimately triggering mitophagy and neuronal necroptosis. However, this effect could be substantially mitigated by PARL overexpression or PGAM5 downregulation, thereby preventing Meth‐mediated neuronal injury.

Collectively, our study demonstrates that Meth downregulates PARL expression, which in turn causes the aberrant cleavage of PINK1 and PGAM5. The increased PGAM5 level ultimately triggers the recruitment of p‐MLKL on the mitochondrial membrane to form pores that disrupt mitochondrial homeostasis, ultimately triggering excessive mitophagy and necroptosis (Figure 12). Noteworthily, PARL overexpression dramatically reversed the Meth‐induced excessive mitophagy and autophagic flux defects in primary neurons and improved cognitive behaviors. Therefore, targeting the PARL‐PGAM5 signaling axis may provide novel insights into potential therapeutic approaches for mitigating Meth‐induced neuronal necroptosis.

## Author Contributions

Xufeng Chen, Jun Wang, Di An, and Chuling Zhang designed the study. Di An, Chuling Zhang, Peng Zhou, Yifei Wang, Sining Meng, Yanlong Chen, Weixiao Xu, and Jiankang Xuan performed the experiments for this work. Di An and Chuling Zhang analyzed data in this study. Jianping Xiong, Jie Cheng, and Rong Gao provided resources and lab management. Di An and Chuling Zhang wrote the manuscript. All authors contributed to the discussion of the study and the revision of this manuscript.

## Conflicts of Interest

The authors declare no conflicts of interest.

## Supporting information


Figures S1‐S3.

Table S1.

## Data Availability

The data presented in the study are included in the article/Supporting Information, and further inquiries can be directed to the corresponding authors.
