# Supplementary material for: Cleaving PINK1 or PGAM5? Involvement of PARL in Methamphetamine‐Induced Excessive Mitophagy and Neuronal Necroptosis
Source: CNS Neurosci Ther. 2025 Feb 27;31(2):e70293. doi: 10.1111/cns.70293 (PMC11865887; doi:10.1111/cns.70293)
Supplement: Supplementary file 1 — Figures S1‐S3. [file CNS-31-e70293-s002.docx]

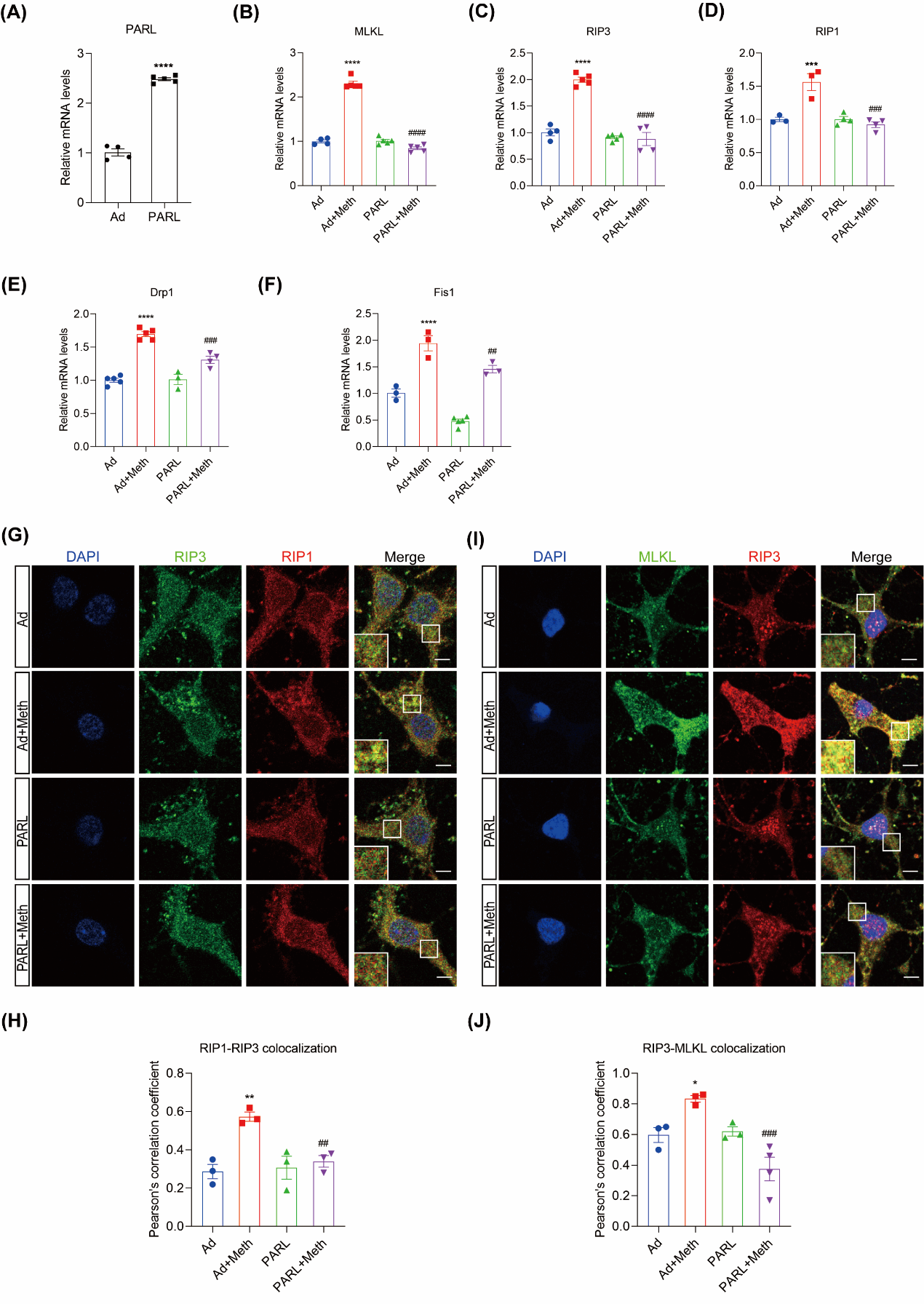


**Supplementary Figure 1:** (A) The PARL overexpression efficiency in neurons was detected by RT-qPCR. (B-F) MLKL, RIP3, RIP1, Drp1 and Fis1 mRNA levels of neurons. (G) RIP3 (green) and RIP1 (red) co-staining. Scale bar=5 μm. (H) Quantitative analysis of colocalization of RIP3 and RIP1. (I) MLKL (green) and RIP3 (red) co-staining. Scale bar=5 μm. (J) Quantitative analysis of colocalization of MLKL and RIP3. ^∗^p < 0.05, ^∗∗^p < 0.01 and ^∗∗∗∗^p < 0.0001 vs. Ad group; ^##^p < 0.01, ^###^p < 0.001 and ^####^p < 0.0001 for the PARL + Meth group vs. Ad + Meth group.


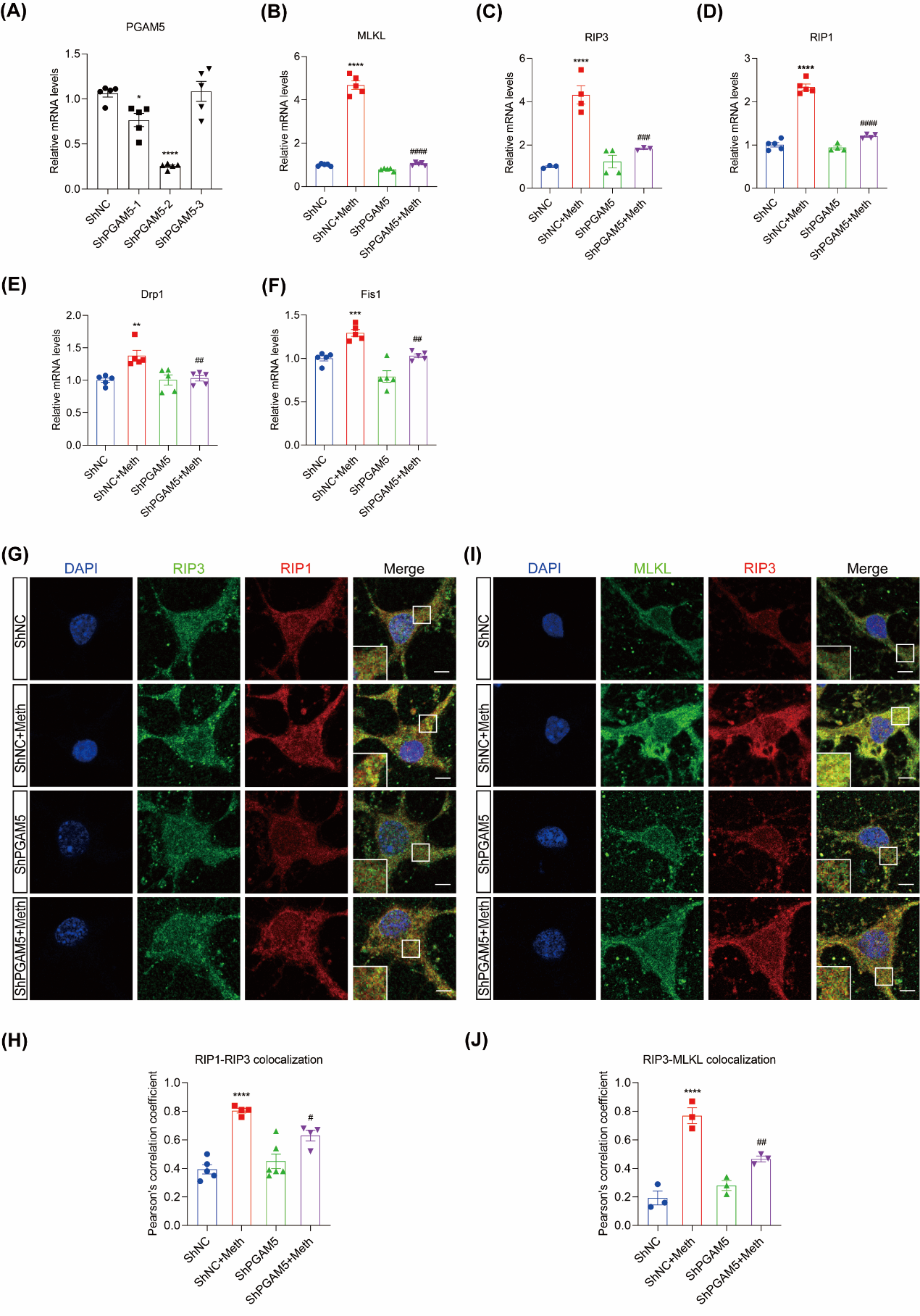


**Supplementary Figure 2:** (A) The PGAM5 knockdown efficiency in neurons was detected by RT-qPCR. (B-F) MLKL, RIP3, RIP1, Drp1 and Fis1 mRNA levels of neurons. (G) RIP3 (green) and RIP1 (red) co-staining. Scale bar=5 μm. (H) Quantitative analysis of colocalization of RIP3 and RIP1. (I) MLKL (green) and RIP3 (red) co-staining. Scale bar=5 μm. (J) Quantitative analysis of colocalization of MLKL and RIP3. ^∗^p < 0.05, ^∗∗^p < 0.01 and ^∗∗∗∗^p < 0.0001 vs. ShNC group; ^#^p < 0.05, ^##^p < 0.01, ^###^p < 0.001 and ^####^p < 0.0001 for the ShPGAM5 + Meth group vs. ShNC + Meth group.


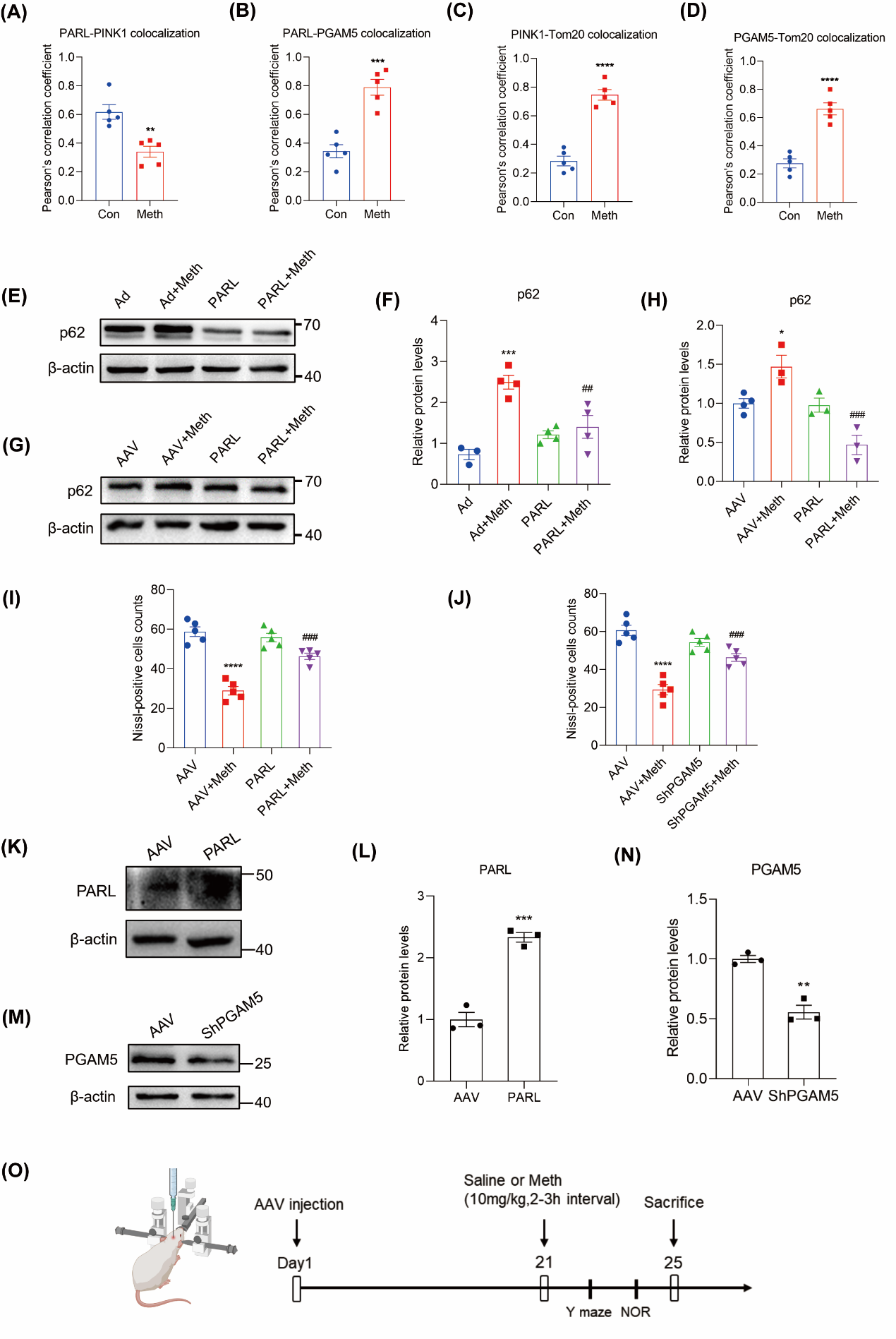
**Supplementary Figure 3:** (A) Quantitative analysis of colocalization of PARL and PINK1. (B) Quantitative analysis of colocalization of PARL and PGAM5. (C) Quantitative analysis of colocalization of PINK1 and Tom20. (D) Quantitative analysis of colocalization of PGAM5 and Tom20. ∗∗p < 0.01, ∗∗∗p < 0.001 and ∗∗∗∗p < 0.0001 vs. Con group. (E) Detection of p62 protein expression by Western blot. (F) Statistical results of the relative expression level of p62 protein. ^∗∗∗^p < 0.001 vs. Ad group; ^##^p < 0.01 for the PARL + Meth group vs. Ad + Meth group. (G) Detection of p62 protein expression by Western blot. (H) Statistical results of the relative expression level of p62 protein. ^∗^p < 0.05 vs. AAV group; ^###^p < 0.001 for the PARL + Meth group vs. AAV + Meth group. (I)

Quantitative analysis of Nissl-positive cells. ^∗∗∗∗^p < 0.0001 vs. AAV group; ^###^p < 0.001 for the PARL + Meth group vs. AAV + Meth group. (J) Quantitative analysis of Nissl-positive cells. ^∗∗∗∗^p < 0.0001 vs. AAV group; ^###^p < 0.001 for the ShPGAM5 + Meth group vs. AAV + Meth group. (K) The PARL overexpression efficiency in hippocampus was detected by Western blot. (L) Statistical results of the relative expression level of PARL protein. ^∗∗∗^p < 0.001 vs. AAV group. (M) The PGAM5 knockdown efficiency in hippocampus was detected by Western blot. (N) Statistical results of the relative expression level of PGAM5 protein. ^∗∗^p < 0.01 vs. AAV group. (O) Graphical representation of the experimental timeline for the mice model. Mice were injected with AAV in the hippocampus 3 weeks before Meth injection.
