# Supplementary material for: Cleaving PINK1 or PGAM5? Involvement of PARL in Methamphetamine‐Induced Excessive Mitophagy and Neuronal Necroptosis
Source: CNS Neurosci Ther. 2025 Feb 27;31(2):e70293. doi: 10.1111/cns.70293 (PMC11865887; doi:10.1111/cns.70293)
Supplement: Supplementary file 2 — Table S1. [file CNS-31-e70293-s001.docx]

The following primers were used:

|  | Forward | Reverse |
| --- | --- | --- |
| PARL | 5′- GGAGGTGGCTCTAAGTGAGG-3′ | 5′- GAGACTTCCGAGCAACAAGGA-3′ |
| PGAM5 | 5′- GGAGGAGGACAGCTACGAGA-3′ | 5′- AACCTTCTGGGGGAAACTGC-3′ |
| PINK1 | 5′- AGCCTTGGGTTCAGCAAACA -3′ | 5′- GCCTCGGTGACAGCTAAGTC -3′ |
| Parkin | 5′- GCTGTCCCAACTCCCTGATT-3′ | 5′- CAGAAAACGAACCCACAGCC-3′ |
| Fis1 | 5′- GTTTGAATACGCCTGGTGCC -3′ | 5′- CAGTCCCCGCACATACTTCA -3′ |
| Drp1 | 5′-GAACCGACAACAGGCAAC-3′ | 5′-GGAACTGGCACATCTAGCA-3′ |
| MLKL | 5′- CTGTGAACTCGGAACCCTGA-3′ | 5′- TACAAGCCTCTAGCTGCCCT-3′ |
| RIP1 | 5′- TCACCAAGAAGAACGGAGGC -3′ | 5′- AAAGCACAATGGCGAAGCTG -3′ |
| RIP3 | 5′- CCCCCAAAGGAATCAGGGAG-3′ | 5′- GGGAAAGGCAGTTCTCGGTT-3′ |
| β-actin | 5′- CGCGAGTACAACCTTCTTGC -3′ | 5′- CCTTCTGACCCATACCCACC -3′ |
